# Lung function and self-rated symptoms in healthy volunteers after exposure to hydrotreated vegetable oil (HVO) exhaust with and without particles

**DOI:** 10.1186/s12989-021-00446-7

**Published:** 2022-01-24

**Authors:** Louise Gren, Katrin Dierschke, Fredrik Mattsson, Eva Assarsson, Annette M. Krais, Monica Kåredal, Karin Lovén, Jakob Löndahl, Joakim Pagels, Bo Strandberg, Martin Tunér, Yiyi Xu, Per Wollmer, Maria Albin, Jörn Nielsen, Anders Gudmundsson, Aneta Wierzbicka

**Affiliations:** 1 Ergonomics and Aerosol Technology, Lund University, 221 00 Lund, Sweden; 2 Lund University, NanoLund, 221 00 Lund, Sweden; 3 Division of Occupational and Environmental Medicine, Lund University, 223 63 Lund, Sweden; 4 Division of Combustion Engines, Lund University, 221 00 Lund, Sweden; 5 School of Public Health and Community Medicine, Institute of Medicine, University of Gothenburg, Gothenburg, Sweden; 6 Department of Translational Medicine, Lund University, Lund, Sweden; 7 Unit of Occupational Medicine, Institute of Environmental Medicine, Karolinska Institute, Stockholm, Sweden; 8 Centre for Healthy Indoor Environments, Lund University, 221 00 Lund, Sweden

**Keywords:** Renewable diesel, Inhalation, Aerosol, Peak nasal inspiratory flow (PNIF), Peak expiratory flow (PEF), Forced oscillation technique (FOT), Symptoms, Pulmonary function, Non-road vehicles, Occupational exposure limits (OELs)

## Abstract

**Background:**

Diesel engine exhaust causes adverse health effects. Meanwhile, the impact of renewable diesel exhaust, such as hydrotreated vegetable oil (HVO), on human health is less known. Nineteen healthy volunteers were exposed to HVO exhaust for 3 h in a chamber with a double-blind, randomized setup. Exposure scenarios comprised of HVO exhaust from two modern non-road vehicles with 1) no aftertreatment system (‘HVO_PM+NOx_’ PM1: 93 µg m^−3^, EC: 54 µg m^−3^, NO: 3.4 ppm, NO_2_: 0.6 ppm), 2) an aftertreatment system containing a diesel oxidation catalyst and a diesel particulate filter (‘HVO_NOx_’ PM1: ~ 1 µg m^−3^, NO: 2.0 ppm, NO_2_: 0.7 ppm) and 3) filtered air (FA) as control. The exposure concentrations were in line with current EU occupational exposure limits (OELs) of NO, NO_2_, formaldehyde, polycyclic aromatic hydrocarbons (PAHs), and the future OEL (2023) of elemental carbon (EC). The effect on nasal patency, pulmonary function, and self-rated symptoms were assessed. Calculated predicted lung deposition of HVO exhaust particles was compared to data from an earlier diesel exhaust study.

**Results:**

The average total respiratory tract deposition of PM1 during HVO_PM+NOx_ was 27 µg h^−1^. The estimated deposition fraction of HVO PM1 was 40–50% higher compared to diesel exhaust PM1 from an older vehicle (earlier study), due to smaller particle sizes of the HVO_PM+NOx_ exhaust. Compared to FA, exposure to HVO_PM+NOx_ and HVO_NOx_ caused higher incidence of self-reported symptoms (78%, 63%, respectively, vs. 28% for FA, *p* < 0.03). Especially, exposure to HVO_PM+NOx_ showed 40–50% higher eye and throat irritation symptoms. Compared to FA, a decrement in nasal patency was found for the HVO_NOx_ exposures (− 18.1, 95% CI: − 27.3 to − 8.8 L min^−1^, *p* < 0.001), and for the HVO_PM+NOx_ (− 7.4 (− 15.6 to 0.8) L min^−1^, *p* = 0.08). Overall, no clinically significant change was indicated in the pulmonary function tests (spirometry, peak expiratory flow, forced oscillation technique).

**Conclusion:**

Short-term exposure to HVO exhaust concentrations corresponding to EU OELs for one workday did not cause adverse pulmonary function changes in healthy subjects. However, an increase in self-rated mild irritation symptoms, and mild decrease in nasal patency after both HVO exposures, may indicate irritative effects from exposure to HVO exhaust from modern non-road vehicles, with and without aftertreatment systems.

**Supplementary Information:**

The online version contains supplementary material available at 10.1186/s12989-021-00446-7.

## Introduction

Exposure to petroleum diesel engine exhaust is known to cause adverse health effects [1–4], and since 2012, diesel engine exhaust has been classified as carcinogenic to humans [5]. Several human exposure studies have linked diesel exposure to acute health effects, among them short-term reduced lung function [6–10], airway inflammatory responses [10–13], irritation symptoms [6, 7, 14, 15], and cardiovascular effects [16–18]. Stricter emission standards in recent years and improved emission reduction techniques have reduced the particle and gas emissions from modern diesel vehicles. At the same time, the use of renewable diesel fuels has increased rapidly in an effort to reduce net CO_2_ emissions. Renewable diesel fuels comes in many heterogenous forms, such as hydrotreated vegetable oil (HVO, often called ‘renewable diesel’) and fatty acid methyl ester (FAME) fuel types (often called ‘biodiesel’) [19]. These types of fuels generally decrease the particulate matter (PM) emissions compared to petroleum diesel [20–23]. However, compared to petroleum diesel the toxicity of these emissions is less evaluated. Only a few controlled human exposure studies of FAME type fuels exist [8, 24], but to the best of the authors’ knowledge there are no such studies on HVO except the co-publications concerning this study from Krais et al. [25] and Scholten et al. [26]. In addition, there is only one study that have investigated the acute effect in humans after exposure to diesel exhaust from vehicles with and without an aftertreatment system (i.e. a diesel particulate filter) [27]. Compared to FAME fuels, HVO is the preferred fuel for full substitution (pure biofuel, B100) of petroleum diesel in non-modified diesel engines due to its greater engine compatibility. With all the known adverse effects of petroleum diesel exhaust from older vehicles, the research focus should be directed to investigate the health effects of realistic exposure concentrations from modern renewable fuels used in vehicles with different exhaust aftertreatment systems.

The exhaust emissions of HVO are, on the one hand, similar to petroleum diesel as the combustion generates carbonaceous PM (“soot”), nitrogen oxides (NO_x_), polycyclic aromatic hydrocarbons (PAHs), etc., but on the other hand, they are different in terms of concentration, particle size and chemical composition [28–31]. For example, HVO can decrease the PM emission by 20–50% in comparison to petroleum diesel [21, 22]. It should be noted that HVO is a paraffinic fuel without any feedstock-derived impurities and is hence more chemically similar to petroleum diesel than FAME-type biodiesels [19, 32]. In contrast, the chemical composition (e.g. oxygen content, carbon chain length, degree of unsaturation) of FAME-biodiesels depends on the feedstock, and considerably affects the physical properties of the particle emissions [29, 33, 34]. The aerosol emission characteristics thus differ for HVO and FAME-type fuels [23, 35], and health effect of FAME-type fuels cannot be generalized to HVO fuels. The solid PM fraction of the exhaust from diesel and HVO is dominated by soot, which can be measured thermo-optically as elemental carbon (EC). A new European Union (EU) occupational exposure limit (OEL) for diesel engine exhaust, measured as EC, of 50 µg m^−3^ [36] will be implemented in 2023 to reduce the exposure of the 3.6 million workers within the member states [37]. Because estimates of the life-time mortality risk from occupational exposure to diesel for cancer alone (not including myocardial infarction or COPD) indicate that exposure levels need to be kept extremely low [38], it is of key importance to identify potential adverse effects of the substitutes. As the substitution of petroleum diesel by renewable diesel is increasing, a considerable number of people will be exposed to its exhaust. It is hence of interest to understand the potential health effects of exhaust exposure from engines running on HVO, while complying with the future OELs.

Exhaust aftertreatment systems are used in diesel vehicles in order to reduce the environmental and health hazardous emissions of PM (both mass and number concentration), CO, NO_x_, and organic compounds such as PAHs. An aftertreatment system can, for example, contain a diesel oxidation catalyst (DOC) that oxidizes CO and organic compounds [39, 40], and a diesel particle filter (DPF) that oxidizes soot particles which removes significant amounts of PM [41]. Hence use of aftertreatment systems should reduce exposure to such emissions and their associated health effects. However, a recent review assessing the effects of DPFs’ use on health impacts in occupational settings did not present conclusive results [1]. It is thus of interest to investigate the health impact from vehicles with different degrees of emission reduction technology.

Due to improved engine operation, modern diesel engines with or without aftertreatment systems generally emit lower concentrations of pollutants [42] and reduce exhaust particle mass and size [43]. Particle characteristics such as size and morphology are of key importance in considering possible health effects, as these characteristics determine where in the lungs the particles will deposit. The deposition pattern in the lung depends on multiple aerosol characteristics and not solely on the respirable PM mass concentration [44].

In the present study we investigate the human health effects from exposure to exhaust from two modern non-road vehicles (wheel loaders). Due to their different engine manufacturing year (2018–2019), they fall under different emission standards and were equipped with (1) no external aftertreatment device, or (2) a DOC in combination with a DPF. The different emissions aftertreatment allowed for a comparison of the exposure to NO_x_ from diesel engines with and without a particulate fraction and other gaseous pollutants. We aimed to evaluate self-rated symptoms, nasal patency, and pulmonary function after exposure to HVO exhaust from modern non-road vehicles that complied with the EU OELs. Additionally, we compared the calculated lung deposition of HVO exhaust particles with the deposition of petroleum diesel from an older light-duty vehicle presented by Wierzbicka et al. in a previous study [15].

## Results

### Main findings

We compared the effects of exposure to exhaust from hydrotreated vegetable oil (HVO) (a renewable diesel fuel) with filtered air (FA). The vehicle without an external aftertreatment system (HVO_PM+NOx_) generated emissions of PM, NO_x_ and organic compounds. The vehicle with an external aftertreatment consisting of a DOC and DPF (HVO_NOx_) emitted NO_x_ with only negligible concentrations of particles and measured organic components (hereafter referred to only NO_x_).

The two exposures to HVO caused mild self-rated irritations symptoms. 44% reported eye irritation symptoms during the HVO_PM+NOx_ exposure. The number of volunteers who reported throat irritation symptoms was a factor 4.5 and 4 higher for HVO_PM+NOx_ and HVO_NOx_ respectively, compared to FA. In comparison to FA exposure, nasal obstruction (lower PNIF) occurred for the HVO_NOx_ exposure. In the first of the following two sections, we describe the exposure aerosol characteristics and the HVO exhaust particle deposition in the airways in relation to diesel exhaust particles. In the second, we describe the health effects in terms of self-rated symptoms and airway function.

### Exposure and lung deposition

#### Exposure aerosol characteristics

**Fig. 1:**
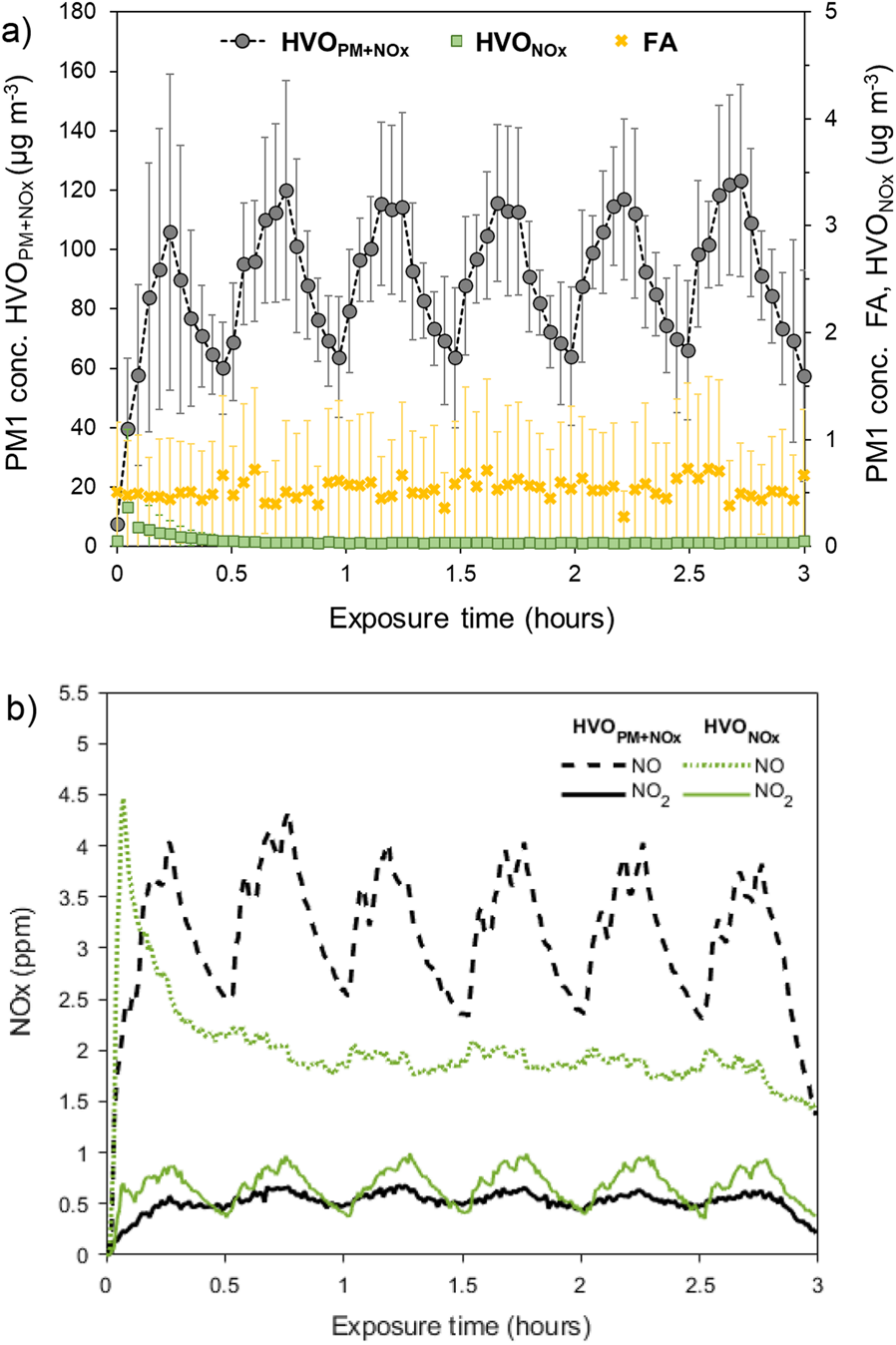
The average values of all the exposures for **a** PM1 mass concentration (measured with SMPS and ρ_eff_), and **b** NO and NO_2_ concentrations. The error bars in **a** correspond to ± 1 std. dev. of all exposures’ averages. The HVO_PM+NOx_ exposure was generated with a wheel loader without an external exhaust reduction system, and HVO_NOx_ with a wheel loader with a diesel oxidation catalyst (DOC) and diesel particulate filter (DPF)

A summary of the average aerosol exposure concentrations and characteristics are presented in Table 1. The average aerosol exposure characteristics during the three exposure scenarios are presented in Fig. 1. The average gravimetric PM1 concentration during the HVO_PM+NOx_ exposures was 93 ± 13 µg m^−3^ and the average particle number (PN) concentration was 3.0 · 10^5^ ± 0.3 · 10^5^ cm^−3^ (Table 1). In contrast, during HVO_NOx_ and FA exposures, the average exposure concentrations of PM1 were ~ 1 µg m^−3^ and PN < 100 cm^−3^ (Table 1). For all exposures, the average particle mass in the range 1–2.5 µm was below 0.1 µg m^−3^ (assessed with the APS), and thus measured PM1 in this study can be approximated to PM2.5. For HVO_PM+NOx_, the elemental carbon (EC) fraction of total carbon was 66 ± 3% corresponding to an average EC concentration of 54 ± 6 µg m^−3^ (Table 1). The two vehicles were operated in a similar load/idle sequence, which is seen as increasing (during load) and decreasing (during idle) PM1 mass (Fig. 1a) and NO concentrations during HVO_PM+NOx_ exposure, and increasing/decreasing NO_2_ levels for the HVO_NOx_ exposures (Fig. 1b).

**Table 1:** Summary of the average aerosol concentrations and characteristics during exposures in the chamber

		HVO_PM+NOx_	HVO_NOx_	FA
Particle phase	PM1 (µg m^−3^) (*Gravimetric)*	93 ± 13	~ 1^a^	~ 1^a^
	PM1 Total Carbon (TC, µg m^−3^)	82 ± 10	< 1	< 1
	*EC/TC (%)*	66 ± 3	–	–
	*OC/TC (%)*	34 ± 3	–	–
	PM1 (µg m^−3^) (*SMPS* and ${\rho }_{eff}$*)*	81 ± 9	0.0 ± 0.0	0.5 ± 0.5
	GMD_mass_ (nm)	114	–	–
	GSD_mass_ (nm)	1.48	–	–
	PN (cm^−3^)	3.0 · 10^5^	9.0 · 10^1^	7.1 · 10^1^
	PN std. dev	0.3 · 10^5^	4.1 · 10^1^	4.3 · 10^1^
	GMD_PN_ (nm)	71	47.2	–
	GSD_PN_ (nm)	1.64	1.46	–
	Surface area (cm^−2^ cm^−3^)	9.5 · 10^–5^	–	–
	Surface area std. dev (cm^−2^ cm^−3^)	1.4 · 10^–5^	–	–
	Particle phase PAHs^b^ (ng m^−3^)	43 ± 3	0.3 ± 0.7	0.2 ± 0.2
Gas phase	Gas phase PAHs^b^ (ng m^−3^)	850 ± 48	97 ± 11	116 ± 29
	Formaldehyde (µg m^−3^)	51 ± 6	< 8	< 8
	Sum BTEX (µg m^−3^)	7.9 ± 2.5	1.7 ± 0.3	1.3 ± 0.1
	VOCs (ppb)	333 ± 69	11 ± 5	< 10
	NO (ppm)	3.4 ± 0.1	2.0 ± 0.1	< 0.001
	NO_2_ (ppm)	0.57 ± 0.04	0.70 ± 0.04	< 0.001
	NO/NO_2_ ratio	5.7	2.9	-
	CO_2_ (ppm)	1344 ± 143	1332 ± 135	813 ± 140

All values are the average of all exposures (n = 5–6) of a given type with ± 1 SD. Full compound analyses of PAHs and BTEX are found in Additional file 1: A and B, respectively

^a^ There were large uncertainties in the gravimetric mass analysis at low/no mass concentrations. The mass concentrations were in the range of the blank filters (− 1 ± 3 µg)

^b^ 33 native and alkylated, 10 oxy- and 17 nitro-PAHs were included in the analysis

The NO and NO_2_ concentrations were comparable for the two HVO exposures (no vehicle had external NO_x_ removal devices, such as selective catalytic reduction [SCR]) and on average below the 8 h OELs. During the HVO_NOx_ exposure, the average NO_2_ was slightly higher and the average NO lower compared to HVO_PM+NOx_ (Table 1). The variations in NO and NO_2_ from the load/idle operation as described above can be seen in Fig. 1b. In addition, NO increased rapidly after the cold start at the beginning of the HVO_NOx_ exposure and decreased shortly thereafter when the diesel oxidation catalyst (DOC) of the vehicle has reached the operating temperature and started to convert NO to NO_2_ more efficiently.

During the HVO_PM+NOx_ exposure scenario (vehicle without aftertreatment) gas-phase organic compounds were quantified, but below the OELs, among them PAHs (889 ± 53 ng m^−3^), BTEX (11.6 ± 3.0 µg m^−3^), and formaldehyde (51 ± 6 µg m^−3^). These emissions were low or below limit of detection (LOD) during the other exposure scenarios. Full PAH (33 native and alkylated, 10 oxy- and 17 nitro-PAHs) and BTEX analyses are presented in Additional file 1: A and B, respectively.

**Fig. 2:**
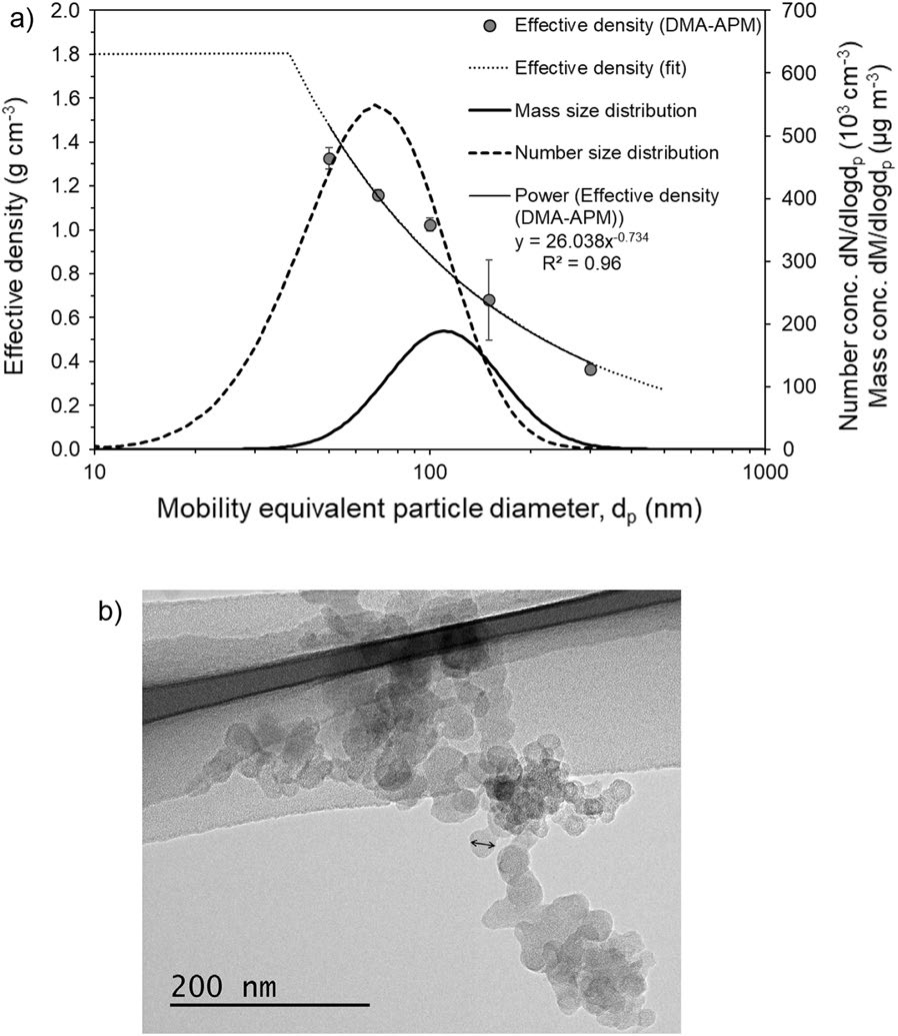
**a** The particle number and mass size distribution together with the average effective density measured with the DMA-APM (± 1 SD). The effective density (${\rho }_{eff}$, g cm^−3^) describes the ratio between measured particle mass and mobility size-derived volume (assuming a sphere). The effective density was measured at 50, 70, 100, 150, and 300 nm as illustrated. The effective density is extrapolated, and below 40 nm the inherent material density of soot (1.8 g cm^−3^) is used. The mass size distribution is derived from the number size distribution and effective density. **b** TEM image of a cluster of particles (soot agglomerates) generated during the HVO_PM+NOx_ exposure, with an arrow showing a primary particle of approximately 25 nm in diameter

The particle number and mass size distribution of HVO_PM+NOx_, together with the effective density of the soot agglomerates, are shown in Fig. 2a. The effective density decreased with increasing mobility size, as the soot agglomerates becomes more open in their structure. The diesel soot power law function described by Park et al. [45] was fitted to the experimental data (Fig. 2a). The mass mobility exponent (D_fm_) was on average 2.3 (where 3 corresponds to perfect spheres). An example of the soot agglomerates generated during the HVO_PM+NOx_ exposure is imaged by transmission electron microscopy (TEM) in Fig. 2b. The average primary particle diameter of the soot agglomerates of HVO_PM+NOx_ was 24.5 ± 7.3 nm and an example is marked in Fig. 2b.

#### Inhaled deposited fraction and dose

**Fig. 3:**
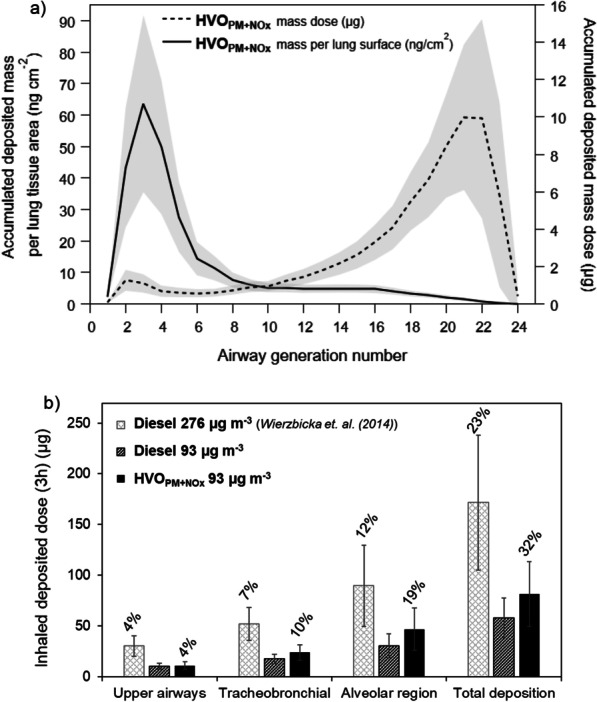
**a** The predicted average accumulated deposited particle dose (gravimetric PM1) in each lung genereation for all test subjects expressed as mass (µg) and mass per lung area tissue (ng cm^−2^) for the HVO_PM+NOx_ 3 h exposure. The shaded area represents the group’s individual variability of ± 1 std. dev. Deposited dose in **a** comprises calculated deposition in the tracheobronchial region (airway generation number 0–15) and alveolar region (gen. 16–24), but not in upper airways. **b** The predicted average accumulated deposited particle dose (µg) in the upper airways, tracheobronchial region and alvolar region as well as the total deposited dose for the HVO_PM+NOx_ exposure. Additionally presented is a comparison to the calculated deposition of petroleum diesel from a previous exposure study with both original (276 µg m^−3^) and reduced mass concentration (93 µg m^−3^) [10]. The number on top of each bar represents the fraction of the deposited mass compared to the total exposure mass concentration. The error bars represent the group’s individual variability of ± 1 std. dev. The deposited fraction (%) is the same for both petrolum diesel aerosols (given only above the first bars) as it is independent on the exposure mass concentration

The average estimated inhaled deposited particle mass from nasal breathing during the HVO_PM+NOx_ exposure in the tracheobronchial and alveolar regions, from airway generation 1 to 24, is presented in Fig. 3a. The calculations were performed with the multiple-path particle dosimetry model (MPPD, [46]). Deposited doses are given as deposited mass (µg) and as deposited mass per lung tissue area (ng cm^−2^). The mass dose was estimated to be largest in the distal airways (airway generations > 10), but when expressed as deposited mass per lung tissue area the largest dose was found in the upper tracheobronchial region (airway generation < 10). No deposition was analyzed for HVO_NOx_ due to low PM and PN concentrations (Table 1).

A comparison is presented in Fig. 3b of the calculated total deposited particle mass doses in the different airway regions in the current study and in a previous petroleum diesel exposure study [6, 15]. The comparison was made with MPPD calculations for both the original diesel mass concentration in the past study (276 µg m^−3^, light grey bars) and with the same mass concentration as in this study (93 µg m^−3^, dark grey bars) using the original aerosol characteristics of the petroleum diesel (MMD 195 nm, GSD 1.65). The deposited mass of the diesel aerosol was higher in comparison to HVO_PM+NOx_, but lower when using the same exposure mass concentrations. The predicted deposited fractions of the HVO_PM+NOx_ aerosol mass (i.e., the fraction of the deposited mass compared to the total inhaled mass concentration) were around 40–50% higher compared to petroleum diesel in the tracheobronchial and alveolar regions. The average accumulated deposited dose in the respiratory tract was 82 µg for HVO_PM+NOx_, corresponding to an hourly average of 27 µg h^−1^. It should be noted that the predicted deposition with the MPPD model might deviate from the actual deposition during exposure, as the measured minute ventilation was higher (median: 15 L min^−1^) than reference values of adult in rest (6 L min^−1^, [47]). This is likely because the tidal volume was measured by breathing through a mouthpiece during a limited period.

The inhaled deposited dose depends on multiple lung parameters (FRC, tidal volume, breathing pattern, etc.). For additional comparison with petroleum diesel, the same deposition model and breathing parameters (oral breathing, [48]) was used as in Wierzbicka et al. [15]. Table 2 presents the deposited HVO_PM+NOx_ particle dose expressed as mass, number and surface area compared to the petroleum diesel exposure (using the original exposure concentrations). Please note the three times higher particle mass concentration in the case of diesel in comparison to HVO when looking at values of deposited dose by mass. The calculated mass (and surface area) deposition fraction was a factor 1.5 higher for HVO_PM+NOx_ than diesel. Additionally, the deposited dose in terms of particle number was similar for HVO_PM+NOx_ and diesel. This is due to the higher calculated deposition fraction of HVO_PM+NOx_ caused by the smaller particle sizes, even though the total exposure particle number concentration was lower (− 23%). The deposited mass fractions differ slightly from the multiple-path particle dosimetry (MPPD) model due to model characteristics, see Additional file 1: C for a comparison of the deposition fraction depending on particle size for the two models.

**Table 2:** The average predicted lung deposition (oral breathing) fractions of PM1 of HVO_PM+NOx_ and petroleum diesel

		HVO (PM1)	Diesel (PM1), Wierzbicka et al. 2014	Unit
Mass concentration (µg m^−3^)		93 ± 13	276 ± 56	
Particle number concentration (µg m^−3^)		3.0 · 10^5^ ± 0.3 · 10^5^	3.9 · 10^5^ ± 0.5 · 10^5*^	
Surface area concentration (µg m^−3^)		9.5 · 10^–5^ ± 1.4 · 10^–5^	3.5 · 10^–4^ ± 0.7 · 10^–4^	
Mass	Deposited fraction	0.40 ± 0.004	0.27 ± 0.01	
	Deposited dose during 3 h exposure	59.4 ± 7.4	118.5 ± 21.6	µg
Number	Deposited fraction	0.52 ± 0.002	0.45 ± 0.03	
	Deposited dose during 3 h exposure	2.6 · 10^11^ ± 3.1 · 10^10^	2.8 · 10^11^ ± 3.5 · 10^10^	Particles
Surface area	Deposited fraction	0.40 ± 0.004	0.27 ± 0.01	
	Deposited dose during 3 h exposure	61.9 ± 7.7	151.9 ± 27.7	cm^2^

The average lung deposition (oral breathing) fractions of PM1 of HVO_PM+NOx_ was calculated with the model presented by Rissler et al. [48] and compared to the calculated deposited doses of petroleum diesel from Wierzbicka et al. [15]. The average mass, particle number and surface area concentrations of the respective exposures are given. All values are presented as mean ± 1 std. dev

*PM0.5

### Health effects

#### Self-rated symptoms

The number of volunteers reporting symptoms from eye, throat, nose, and chest during the exposures are shown in Table 3 (symptoms VAS scores in Additional file 1: D). Exposure to HVO (HVO_PM+NOx_ and HVO_NOx_) caused significantly higher incidences of reported symptoms compared to FA (78%, 63% vs. 28%, *p* < 0.03 for both). The proportion of volunteers who reported throat irritation was a factor 4.5 and 4 higher for HVO_PM+NOx_ and HVO_NOx_, respectively, compared to FA. The difference was statistically significant for HVO_PM+NOx_ (*p* = 0.011) and with borderline significance for HVO_NOx_ (*p* = 0.062). The proportion of reported eye irritation symptoms was around a factor 2.5 higher for HVO_PM+NOx_ compared to FA with a borderline significance (*p* = 0.07). No volunteers reported chest tightness during the FA exposure, while a few individuals did so during the HVO_PM+NOx_ and HVO_NOx_, respectively. However, it should be noted that the reported symptom scores were generally low (mostly below 10 in a 0–100 VAS) for all categories.

**Table 3:** Descriptive table of the reported symptoms during each exposure scenario

	Any reported symptom			Eye^a^			Throat^b^			Nose^c^		Chest^d^	
	Yes/total	%	P (${\chi }^{2}$- test)	Yes/total	%	P (${\chi }^{2}$- test)	Yes/total	%	P (${\chi }^{2}$- test)	Yes/total	%	Yes/total	%
FA	5/18	28	–	3/18	17	ref	2/18	11	ref	1/18	6	0/18	0
HVO_NOx_	12/19	63	0.031	7/19	37	0.27 ^e^	8/19	42	0.062^e^	2/19	11	1/19	5
HVO_PM+NOx_	14/18	78	0.003	8/18	44	0.07	9/18	50	0.011	5/18	28	3/18	17

Descriptive table of the number of volunteers reporting any type of symptoms, symptoms categorized by type, and χ^2^-tests for any reported symptoms, eye and throat symptoms. The ${\chi }^{2}$-tests are compared to the FA exposure. χ^2^-tests were not performed for nose and chest symptoms due to the low number of reported symptoms. The number of subjects was 19 for HVO_NOx_ and 18 for FA and HVO_PM+NOx_

^a^Itching, running and/or sore eyes. ^b^Sore/dry/irritated throat. ^c^Running nose and/or nose congestion. ^d^Chest tightness/breathlessness. ^e^*p* values obtained from Fisher exact test

#### Effects on airway function

##### Peak nasal inspiratory flow (PNIF) and peak expiratory flow (PEF)

**Fig. 4:**
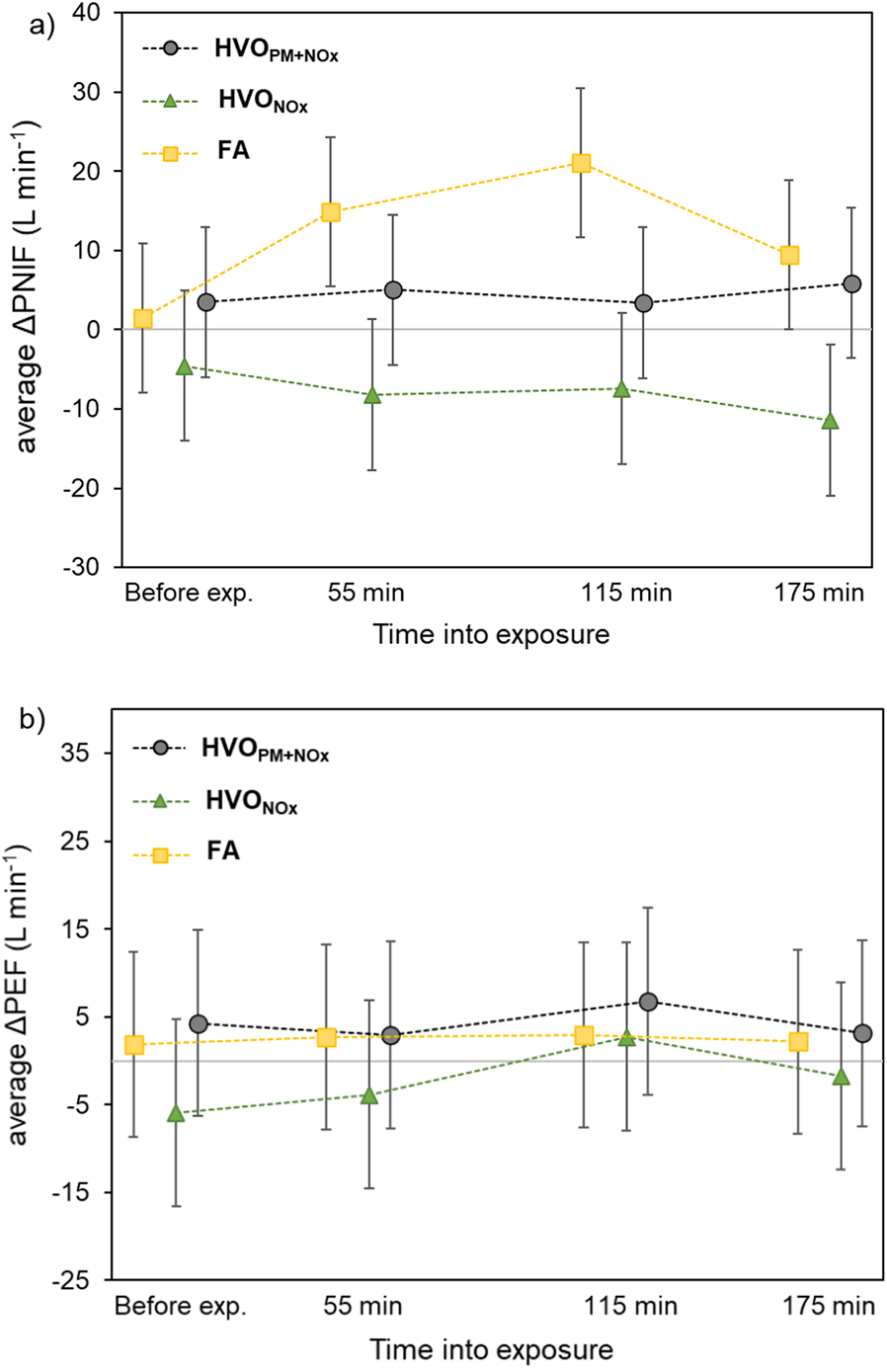
The average changes (exposure order corrected) in **a** PNIF, and **b** PEF (L/min) during the exposures compared to the measurement before exposure. The change in PNIF and PEF from before exposure (ΔPNIF, ΔPEF) are estimated from linear mixed model after adjusting for exposure order. The measurements for all exposures are made at exactly the same time points (i.e., before exposures and at 55, 115, and 175 min into the exposure). The visible shift on the graph between exposures was applied to clearly show the values. The error bars represent the 95% confidence interval (CI)

The changes in ΔPNIF and ΔPEF at each time point during the exposure are shown in Fig. 4, and absolute values are presented in Additional file 1: E. For both PNIF and PEF, there was an increasing trend throughout the FA exposure while no such increase was seen for neither of the two HVO exposures. The differences between average changes in PNIF and PEF measurements (ΔPNIF and ΔPEF) during the two HVO exposure scenarios compared to FA are presented in Table 4. Although no decrease in absolute PNIF values was found for the HVO exposures, we observed a statistically significant decrement in ΔPNIF during HVO_NOx_ exposure compared to the FA exposure (− 18.1 L min^−1^, *p* ≤ 0.001), and a borderline significant decrement during HVO_PM+NOx_ exposure (− 7.4 L min^−1^, *p* = 0.08). No difference in ΔPEF was found between the HVO exposures and FA.

**Table 4:** The average changes in ΔPNIF and ΔPEF (L min^−1^) during each exposure scenario and compared to the FA exposure

	PNIF			PEF		
	Estimated mean (95%CI)	Beta (95% CI)	*p* value	Estimated mean (95%CI)	Beta (95% CI)	*p* value
FA	10.3 [4.1, 16.6]	Ref	ref	2.2 [− 5.1, 9.5]	Ref	ref
HVO_NOx_	− 7.7 [− 14.4, − 1.1]	− 18.1 [− 27.3, − 8.8]	< 0.001	− 2.5 [− 10.2, 5.3]	− 4.6 [− 15.6, 6.3]	0.40
HVO_PM+NOx_	2.9 [− 3.4, 9.1]	− 7.4 [− 15.6, 0.8]	0.08	4.7 [− 2.4, 11.9]	2.5 [− 7.1, 12.2]	0.60

Estimated average changes in ΔPNIF and ΔPEF (L min^−1^) during each exposure scenario (estimated mean) and differences between the two HVO exposures and FA exposure (beta). The beta values (L min^−1^) and significance (*p* value) are based on the linear mixed model with exposure order correction. Values within brackets are the 95% CI

##### Spirometry

The result of the FVC (forced vital capacity), FEV_1_ (forced expiratory volume in one second) and FEV_1_/FVC (in L, z-score and as % of predicted) is presented in Additional file 1: F. FEV_1_ and FVC showed minimal and statistically insignificant differences after all exposure scenarios (Additional file 1: F1). A minimal (from 0.81 to 0.82) but statistically significant (*p* < 0.05) increase in mean FEV_1_/FVC was found after the HVO_NOx_ exposure, however, no corresponding change in related parameters were found to support an adverse effect. Compared to FA, no significant changes were found after the HVO exposures (Additional file 1: F2).

##### Oscillometry parameters

The results of the oscillometry parameters reflecting reactance (X_5_, A_X_, F_RES_) and resistance (R_5_, R_19_) are presented in Table 5. There was no statistically significant difference before and after any exposure for any parameter (median). Weak statistical evidence (*p* = 0.084) was found for a decrease in reactance (X_5_) after HVO_NOx_; however, similar trends were not seen for the related parameters of A_X_ and F_RES_ which downplays the probability of a physiological effect on the lung. Some volunteers had baseline oscillometry values deviating from the normal range [49, 50] but with normal spirometry measures; thus, the volunteers were further categorized into a “typical” and “atypical” group based on their oscillometric measures (Additional file 1: G). The atypical group showed a higher proportion of having a history of symptoms and atopy (80% vs. 54%, *p* < 0.05). They were hypothesized to be more sensitive to pollutants and have a different lung reaction to the exposures than the typical group. However, no significant interactions between the typical/atypical groups and PNIF or PEF were found. Neither were any statistically significant changes found for any oscillometry parameters of the atypical/typical groups after the HVO exposures in comparison to FA.

**Table 5:** The average lung reactance (X_5_, A_X_), resistance (R_5_, R_19_, R_5–19_) and resonant frequency (F_RES_) before and after exposure and the paired test

	Exposure	Before exposure		After exposure		Related-samples Wilcoxon Signed Rank Test
		Median (25%, 75%)	Mean (Std. dev.)	Median (25%, 75%)	Mean (Std. dev.)	*p* values
R_5_ (cmH_2_O s L^−1^)	FA	3.56 (3.28, 4.16)	3.66 (0.87)	3.86 (3.17, 4.31)	3.82 (1.07)	0.215
	HVO_NOx_	3.75 (2.86, 4.74)	3.98 (1.33)	3.87 (3.13, 4.86)	4.17 (1.67)	0.494
	HVO_PM+NOx_	3.61 (3.1, 4.68)	4.06 (1.67)	3.6 (2.93, 5.22)	4.10 (1.84)	0.845
R_19_ (cmH_2_O s L^−1^)	FA	3.09 (2.63, 3.77)	3.11 (0.73)	3.24 (2.67, 3.71)	3.20 (0.71)	0.286
	HVO_NOx_	3.12 (2.56, 3.96)	3.21 (0.74)	3.33 (2.68, 3.88)	3.28 (0.83)	0.355
	HVO_PM+NOx_	3.14 (2.61, 3.82)	3.17 (0.86)	3.03 (2.54, 3.97)	3.26 (1.00)	0.215
R_5–19_ (cmH_2_O s L^−1^)	FA	0.38 (0.2, 0.72)	0.54 (0.51)	0.51 (0.16, 0.68)	0.63 (0.69)	0.5
	HVO_NOx_	0.79 (0.18, 1.11)	0.77 (0.80)	0.72 (0.24, 0.98)	0.88 (1.08)	0.212
	HVO_PM+NOx_	0.49 (0.22, 1.07)	0.89 (1.05)	0.5 (0.27, 1.15)	0.84 (1.04)	0.948
X_5_ (cmH_2_O s L^−1^)	FA	− 1.19 (− 1.34, − 1.1)	− 1.25 (0.40)	− 1.16 (− 1.38, − 0.99)	− 1.35 (0.71)	0.306
	HVO_NOx_	− 1.21 (− 1.45, − 0.88)	− 1.3 (0.65)	− 1.22 (− 1.53, − 1.1)	− 1.49 (1.02)	0.084
	HVO_PM+NOx_	− 1.15 (− 1.86, − 1.07)	− 1.58 (1.12)	− 1.19 (− 1.51, − 0.99)	− 1.43 (0.96)	0.102
A_X_ (cmH_2_O L^−1^)	FA	5.9 (4.16, 8.72)	7.44 (5.00)	5.63 (2.75, 8.79)	7.85 (6.66)	0.913
	HVO_NOx_	5.99 (3.34, 14.13)	9.24 (7.91)	6.31 (3.19, 13.14)	10.73 (13.13)	0.658
	HVO_PM+NOx_	6.33 (4.26, 15.31)	12.14 (15.50)	5.52 (3.44, 13.97)	12.00 (17.05)	0.586
F_RES_ (Hz)	FA	15.38 (13.59, 19.47)	16.28 (3.88)	15.64 (12.9, 18.88)	15.93 (4.41)	0.528
	HVO_NOx_	14.88 (12.91, 23.66)	17.36 (5.90)	14.88 (12.97, 22.96)	17.61 (5.98)	0.494
	HVO_PM+NOx_	16.22 (12.28, 22.05)	17.64 (5.91)	14.98 (13.56, 19.71)	17.58 (7.18)	0.744

## Discussion

We present the first controlled human chamber exposure to exhaust from the renewable diesel fuel hydrotreated vegetable oil (HVO) assessing the effects on airway function and self-rated symptoms. Modern non-road vehicles with or without an aftertreatment system were used to investigate the health effects from two different but realistic exposures: (1) PM combined with NO_x_ and, (2) NO_x_ alone, both of which were compared to filtered air (FA) exposure. The exposure levels were designed to be close to, but below the current EU 8-h OELs for NO, NO_2_, BTEX and PAHs (naphthalene and benzo(a)pyrene), and the future EU OEL for elemental carbon (EC, from 2023).

We found that exposures to HVO exhaust (both with and without the particle fraction) caused mild irritation symptoms compared to FA. Additionally, the observed PNIF patterns indicate that nasal obstruction occurred during both HVO exposures compared to FA. However, no overall changes in pulmonary function measured as PEF, spirometry or forced oscillation technique (FOT), were observed after 3 h of exposure to HVO exhaust. Our findings indicate that exposure to HVO exhaust from modern non-road vehicles at relatively low exposure levels (below the future OELs) during a short period (3 h) can cause irritative symptoms.

### Self-rated symptoms

Exposure to HVO_PM+NOx_ caused a significantly higher number of reported symptoms compared to FA, and some evidence of a similar trend for symptoms of eye and throat irritation (Table 3). In addition to PM and NO_x_, the HVO_PM+NOx_ exposure also contained low concentrations of VOCs such as formaldehyde, BTEX and PAHs, which were not detected for HVO_NOx_. Formaldehyde exposure is a known irritant, causing eye and respiratory tract irritation [51], however, strong responses are generally found at much higher concentrations (> 200 µg m^−3^, [15, 52]) than used in this study (HVO_PM+NOx_: 51 ± 6 µg m^−3^). The mild symptoms of irritation in this study could hence be an effect of the formaldehyde, despite the low concentration, in combination with the other VOCs and the PM. However, the effect of NO or NO_2_ alone cannot be disregarded since there were symptoms reported for the HVO_NOx_ exposure as well but to a lower extent. Symptoms similar to the ones observed in this study have earlier been reported by Mudway et al. [14] who found nasal, throat, and eye irritation as well as bronchoconstriction in healthy volunteers exposed to petroleum diesel at levels similar to the ones used in this study.

### Airway function

Different patterns in PNIF values were found for the HVO and FA exposures (Fig. 4). In contrast to FA exposure, PNIF did not increase during exposure to the HVO exhaust, hence indicating a nasal obstruction during the two HVO exposures. The lack of an increase in PNIF was seen already 55 min into the exposure (Fig. 4). Exposure to PM2.5 have been suggested to weaken the barrier junctions in the nasal epithelial cells, increasing the risk for nasal symptoms and diseases such as rhinitis [53]. However, the decrements of ΔPNIF were larger during the particle-free HVO_NOx_ exposure than during HVO_PM+NOx_ in comparison to FA (Table 1). As the HVO_NOx_ exposure did not contain any PM fraction (PN < 100 particles cm^−3^, PM ~ 1 µg m^−3^), the effects are attributed to the NO and NO_2_ exposure. In addition, the larger impact on the nasal patency may be related to the NO_2_ rather than the NO concentration since NO was lower during the HVO_NOx_ exposure than during HVO_PM+NOx_. The 3 h average NO_2_ concentration was similar for the two HVO exposures but fluctuated more for HVO_NOx_ and this caused short periods with higher concentrations (Fig. 1b). However, the impact of NO_2_ on nasal patency is not known, and the uptake of NO_2_ generally occurs deeper down in the lungs and causes effect on the small airways [54] and asthma-related respiratory effects (reviewed in [46]). In this study no overall changes in lower airway function (assessed with PEF, spirometry and FOT) were seen, while, for example, reduction in PEF has been reported after petroleum diesel exhaust exposures of both higher [6] and lower [9] PM and NO_x_ exposure concentrations. However, in studies with NO_2_ exposures alone at similar concentrations as in this study (1–3 h, 0.1–4 ppm), NO_2_ has not caused any significant effects on lung function (assessed by spirometry) in healthy subjects [56–59]. On the other hand, Wooding et al. reported a larger impairment in FEV1 after allergen co-exposure with particle depleted diesel exhaust (19 µg m^−3^, 0.15 ppm NO_2_) compared to full diesel exhaust (292 µg m^−3^, 0.05 ppm NO_2_) [60]. This was attributed to the higher NO_2_ exposure (0.15 ppm), and no protective effects of decreasing the PM mass was found. However, the particle removal method in Wooding et al. was carried out by passing an air-cleaner consisting of a mechanical pre-filter and an electrostatic precipitator (in contrast to the DOC + DPF used in this study). Changes in the organic fraction of particles and gases by oxidants formed in the electrostatic precipitation process were not discussed.

Another cause for the increased nasal obstruction after the two HVO exposures could potentially be due to local or pulmonary vasodilation induced by the NO exposure. NO is a known pulmonary and systemic vasodilator, and when clinically administered it causes preferential pulmonary vasodilation in the distal lung, which is used, for example, to treat hypoxemia and acute respiratory distress syndrome (5–80 ppm) [61, 62]. However, if the vasodilative effect have an impact in the upper airways such as the nasal route requires further study. In this study, as the decrement in PNIF (compared to FA) was lower for HVO_PM+NOx_, which contained higher NO than HVO_NOx_, we cannot attribute the nasal obstruction solely to NO. In addition, we cannot exclude that there is an interaction effect of the PM and gases, as the changes in nasal patency were less pronounced during HVO_PM+NOx_ than HVO_NOx_.

The temporary changes in measured airway functions were small and unlikely to have clinical importance in healthy persons from short-term exposure. Nevertheless, we cannot exclude a risk from either short-term or long-term exposure on more sensitive persons, for example older people or those with pre-existing lung or cardiovascular disease. The long-term effect of renewable diesel exhaust is unknown, but the short-term responses in this study indicate that even exposure below the future OELs is not completely without risk for negative health effects. Reduced PNIF is not a measure of lung function, but clinically a long-term nasal obstruction caused by occupational exposure could be considered indicative for the development of irritation asthma [63].

It is possible that the short exposure of 3 h does not cause sufficient effect to be detected by spirometry, as restrictive breathing patterns have been detected with spirometry after rather long-term exposure to higher diesel exhaust concentrations (6 months, 282.3 µg m^−3^ PM2.5, [64]). This highlights the need for more sensitive analysis methods due to the ethical considerations limiting the exposure concentration and length. In previous studies, oscillometry measurements found early manifestations of lung disease before these were measurable with spirometry [65, 66]. One study using oscillometry measurements found increased resistance and reactance correlated to acute diesel exhaust (NO_2_ an CO) exposed symptomatic patients [67], when standard spirometry showed no change in pulmonary function. In COPD patients, short-term exposure to traffic pollution have shown an increased airway resistance (R_20_) and a decrease in FVC [68]. In addition, compared to spirometry, oscillometry measurements better detect changes in the peripheral airways [69], where the majority of the inhaled PM1 is predicted to deposit (Fig. 3). The subjects with baseline oscillometric values just outside the normal range (“atypical group”, Additional file 1: G) were hence hypothesized to have a different lung reaction than persons within the normal range. Differences between the typical/atypical groups were investigated for oscillometric parameters, PNIF and PEF but due to the small sample size, no clear conclusions can be drawn. The subjects with atypical oscillometry measures showed a higher proportion of having a history of symptoms and atopy (80% vs. 54%, p < 0.05) in their initial medical assessment, which indicates that they may be more sensitive to pollutants and allergens. However, studies with larger numbers of subjects need to be carried out in order to draw any conclusions. It should also be noted that this group did not show any indications of anomalies in the spirometry similar to observations previously reported [65–67] and for future controlled exposure studies, the oscillometry measurement may increase the possibility to investigate small differences in lung function. In addition, oscillometry may potentially be valuable in the assessment of lung function effects related to occupational exposures for early detection and disease prevention.

### Aerosol characteristics, deposited dose, and occupational exposure limits

The predicted total deposited mass dose of HVO_PM+NOx_ during the 3 h exposure (82 ± 32 µg, Fig. 3b) was comparable to the hourly mass dose for people working outdoor in relatively polluted cities during a similar time interval (50 µg PM2.5 h^−1^, [70]). Diesel exhaust is dominated by particles below 1 µm [28, 71, 72] and the mass observed between PM1 and PM2.5 in this study was insignificant (below 0.1%). Therefore, the PM1 exposures in this study can be compared to PM2.5 diesel exhaust exposures in other studies. Compared to a previous exposure study on petroleum diesel [15], the HVO particles generated by the modern diesel engine in this study had a smaller mobility size which caused higher deposition fractions in terms of mass, surface area and number. The difference in deposition is due to the higher calculated deposition fraction (Additional File C) of smaller particles which dominated HVO_PM+NOx_ emissions (MMD 108 nm) in comparison to petroleum diesel (MMD 195 nm). It means that in the case of HVO_PM+NOx_, two times more particles are predicted to deposit due to their smaller size in comparison to the compared petroleum diesel particles.

Modern diesel engines utilize improved combustion parameters of, for example, increased fuel injection pressure and nozzle design, which reduce the size (but not necessarily the number concentration) of the soot particles, which in turn reduces the soot mass emissions [43]. As the upcoming OEL for the EC from diesel engines is only expressed as mass (50 µg EC m^−3^, from 2023 in EU), it may be more efficient in mitigating the inhaled and deposited dose of older diesel engine emissions, but not necessarily as efficient in reducing the deposited dose from renewable fuels and modern diesel engines without DPFs. For example, even though the PM1 mass concentration (93 µg m^−3^) was 3 times lower for HVO in this study, compared to the previous exposure study to petroleum diesel [15] with PM1 276 µg m^−3^, the average deposited mass (Table 2) was only 50% lower. Despite the lower PM1 mass, the number concentration was similar and the surface area even higher due to the reduced particle size. OELs in terms of particle number concentration can hence be more efficient in reducing the exposure to particle emissions from renewable fuels and modern diesel engines similar to the ones used in this study. The particle size distributions and number concentrations need to be assessed in exposure studies and not only the mass in order to understand the deposition and dose dependent effects.

Even though the EU emission standards are continuously becoming more stringent with lower allowed exhaust emissions, modern non-road vehicles lacking full emission aftertreatment systems, like the two vehicles used in this study, will still be in use and pose a risk for hazardous exposure. No vehicle in this study had an external NO_x_ reduction unit, and the implementation of one could potentially have reduced the NO_x_ emissions and related health effects. Krais et al. [25] found a small increase in lipid peroxidation (urinary 4-HNE-MA) after the HVO_PM+NOx_ exposure, while Scholten et al. [26] found no genotoxic indications from any of the exposures. From the results in this short-exposure study, we cannot exclude the potential risk of short- and long-term effects from exposure to HVO exhaust, with and without the particle fraction, from modern non-road vehicles that comply with the latest emission standards and the future OELs.

### Limitations

This study had certain practical, instrumental, and ethical limitations. The study was randomized but exposure order was not fully balanced due to practical reasons. However, to compensate for possible first exposure biases, a mock-up exposure before the first real exposure was conducted as well as the imbalanced exposure order was adjusted for in the statistical analysis. The study was nominally double-blinded, and while blinding was effective for personnel performing medical assessment and analysis, we did not test to what degree it was effective for test participants. Some participants initially experienced moderate smell during the HVO exposures, but not during the FA exposure. However, generating diesel exhaust without any smell is not possible, and the study is hence comparable to other reported diesel exposure studies. The study population was recruited locally from university networks and does not reflect the average Swedish population or occupational groups with engine exposures (for instance, median age was relatively low: 29 years, range 20–55). Due to ethical considerations, only a single short-term exposure with relatively low exposure levels was possible. However, the same exposure protocol, methodology, and a similar number of healthy volunteers was studied previously by our group in an exposure study of diesel exhaust (and noise) where we were able to clearly demonstrate short-term effects, proving that the applied methodology is suitable for detection of measurable effects [6, 15, 73]. We did not predesignate primary endpoint and recognize that we have performed multiple comparisons with several endpoints that increases risk of Type I error. A primary endpoint was not feasible to determine beforehand based on available evidence as this was the first human exposure study on HVO and previously unexplored endpoints were included (e.g. oscillometry). The breathing pattern was not possible to monitor continuously, but only to measure in proximity to the exposure sessions. The tidal volume was measured by FOT while breathing through a mouthpiece, which cause an increase in tidal volume, and followingly the deposited doses with the MPPD model might be overestimated.

## Conclusion

We investigated the effects on airway function after exposure to exhaust from HVO (renewable diesel) from modern vehicles with and without an aftertreatment system in comparison to filtered air. The vehicles were manufactured in 2019 and complied with the current non-road engine EU emission standards. The exposure levels were kept below the EU OELs. Mild irritations symptoms (self-rated) were reported during the two HVO exposures, both with and without the particle fraction, however a slightly higher incidence number was found during the whole HVO exhaust exposure from the vehicle without an aftertreatment system (HVO_PM+NOx_). The data also suggested that some individuals might be affected by exposure to HVO exhaust from modern work vehicles below the future EU OELs. Compared to older diesel exhaust exposures, the calculated deposited fraction in the respiratory tract of HVO exhaust PM was higher, in terms of mass, number and surface area. The increase in deposition was due to the smaller soot particle size.

In this study, the focus was on nasal patency and pulmonary function assessments. However, to understand the full potential health effects of HVO exposures, additional analyses of the inflammatory and cardiovascular effects need to be performed. Additionally, only the effects of short-term exposure to HVO exhaust were explored in this study. This means that any conclusions about the long-term effects and effects on potentially sensitive groups need to be addressed in future studies.

Although the HVO fuel is more sustainable from a climate perspective, our study indicates that from a health perspective, the exposure levels need to be as carefully controlled as they are for petroleum diesel.

## Method

### Study design

In total 19 volunteers (9 f /10 m, age 20–55 years) were exposed to the two types of engine emissions and particle free air during three separate 3 h long sessions at least one week apart. The exposure scenarios discussed in this publication were: (1) emissions from a wheel loader without exhaust aftertreatment operated with HVO (HVO_PM+NOx_), (2) emissions from a wheel loader with an aftertreatment system operated with HVO (HVO_NOx_), and (3) filtered air (FA). We would like to point out that the whole study also included a fourth exposure scenario, namely, exposure to aerosolized dry NaCl. The results of this will be presented separately. The study was designed to analyze the effect of the two HVO exposures in comparison to filtered air. The exposures took place in a 22 m^3^ stainless steel chamber with controlled relative humidity, temperature, and ventilation. A maximum of four participants were exposed, seated quietly, at the same time. The study design was double-blind and with randomized exposure order. The noise level varied from 42–46 dB for all exposures and was caused by the background noises from the ventilation and instruments surrounding the chamber. Some participants initially experience moderate (on average maximum 3 out of a scale of 10) smell during both HVO exposures, but less during FA exposure. Only researchers involved in generation of particles and their monitoring knew what type of exposure was carried out in a given day and did not perform any medical assessments. The exposure order was randomized but not fully balanced due to practical limitations during the commencement of the study, full details of exposure order are given in Additional file 1: H and I. To avoid possible biases of first-time exposure, all participants went through a shorter mock-up of the exposure session during the baseline exam (> 1 week before first exposure) to perform the baseline examinations and familiarize the subjects with the exposure chamber and all medical procedures. In addition, the imbalanced exposure order was accounted for in the statistical analysis. Out of 24 possible exposure orders, 9 were used (details in Additional file 1: I). All exposures took place on Tuesdays, Wednesdays and Thursdays, between 9 and 12 a.m., with a minimum of one week between each exposure. The study was intentionally performed during the fall in Sweden to ensure minimal levels of allergens in the outside air (and the study participants were not allowed to take allergy medication). Before and immediately after each exposure, the participants went through medical examinations that included measurements of standard pulmonary function by spirometry and respiratory mechanics by the forced oscillation technique (FOT). Self-rated symptoms, PEF (peak expiratory flow) and PNIF (peak nasal inspiratory flow) were measured four times: one time before and three times during the exposure (Table 6).

**Table 6:** Scheduling and time points of the reported measurements and self-rated symptoms

Item	Before exposure	During exposure (time after exposure start in minutes)*			Immediately after exposure
Time point	1	2	3	4	5
Self-rated symptoms	X	35 min	95 min	155 min	
Peak Nasal Inspiratory Flow (PNIF)	X	55 min	115 min	175 min	
Peak Expiratory Flow (PEF)	X	55 min	115 min	175 min	
Spirometry	X				X
Forced Oscillation Technique (FOT)	X				X

*The exposure lasted 3 h (180 min)

#### Study population

Volunteers were recruited via Lund University online channels and posters. Of the 25 volunteers who underwent the initial medical examination (spirometry, medical history), 19 fulfilled the inclusion criteria and were consecutively selected for the study. The inclusion criteria were the following: men or non-pregnant women; 20–65 years old; no symptoms or diagnosis of lung disease or asthma; normal standard ECG reading; no allergy or cardiovascular medication; non-smoker the last three years. All except one of the selected participants underwent all three exposures. One person only participated in the exposure to HVO_NOx_. Characteristics of the study group are summarized in Table 7 and in detail in Additional file 1: H. Out of the 7 former smokers, only one was considered a previous heavy smoker (smoked up to 2 packages of cigarettes a day for 10 years) but had been a non-smoker the past 30 years. The smoking habits of the other 6 former smokers varied between < 1 up to 35 cigarettes a week for 1–5 years, and all had been smoke-free the last 4 years. No subjects regularly used any medication except combined oral contraceptive pill (1/19), vitamin C (1/19) and vitamin D (1/19). The study was approved by the Swedish Ethical Review Authority (registration no. 2019-03320) and performed in accordance with the Declaration of Helsinki.

**Table 7:** The participants’ medical history and results from the initial medical examinations before commencement of the study

		Subjects (N = 19)
	Age (median, min–max)	29 (20–55)
	Female (N, %)	9 (47%)
	Previous smoker (N, %)*	7 (37%)
Medical history	History of any symptoms last 12 months (N, %)	7 (37%)
	Eye symptoms (N, %)	1 (5%)
	Nasal symptoms (N, %)	3 (16%)
	Dry cough (N, %)	0
	History of chronic bronchitis symptoms (N, %)	0
	History of bronchial hyperreactivity symptoms (N, %)	5 (26%)
History of childhood atopy	Atopic dermatitis/Childhood eczema (N, %)	3 (16%)
	Allergic rhinitis/Hay fever (N, %)	1 (5%)
	Urticaria (N, %)	1 (5%)
	Physician-diagnosed asthma during childhood (N, %)	1 (5%)
Atopy	Phadiatop positive (N, %)	7 (37%)
Baseline spirometry (prior to bronchodilation)	FVC % pred. (median, min–max)	92 (70–106)
	FEV_1_% pred. (median, min–max)	94 (73–108)
	FEV_1_/FVC % pred. (median, min–max)	100 (90–108)
Baseline spirometry (after bronchodilation)	FVC % pred. (median, min–max)	95 (72–107)
	FEV_1_% pred. (median, min–max)	97 (79–109)
	FEV_1_/FVC % pred. (median, min–max)	100 (90–112)

^*^All test subjects were currently non-smokers

FVC = forced vital capacity, FEV_1_ = forced expiratory volume in one second

### Aerosol generation

The exposures took place in a 22 m^3^ stainless steel chamber with an air exchange rate of 4 exchanges per hour. The supply air used for dilution was filtered from particles with a HEPA (high-efficiency particulate absorbing) filter and from gases with an active carbon filter. The temperature was kept at 26 ± 1 °C and the relative humidity at 33 ± 4%.

#### Renewable diesel exposure

The renewable diesel exhaust exposure scenarios were generated with two types of modern off-road diesel vehicles with different net power (kW). Both vehicles were manufactured in 2019 (but the smaller vehicle's engine was manufactured in 2018) and complied with the current EU emission legislation. The smaller vehicle had a 1.6 L 3-cylinder diesel engine, net power 23 kW, and followed emission standard Stage IIIa (2007). The larger vehicle had a 2.9 L 4-cylinder diesel engine, net power of 55.4 kW, and followed Stage V (2019). The smaller vehicle was not equipped with any external exhaust aftertreatment (hereafter denoted HVO_PM+NOx_), while the larger vehicle was equipped with a diesel oxidation catalyst (DOC) and a diesel particulate filter (DPF) (hereafter denoted HVO_NOx_).

The vehicles were started immediately after the study subjects had settled in the exposure chamber and were kept running until the end of exposure. The operation of the vehicles always started with a cold start (outside temperature 5.5 ± 4.1 °C), thus cold start was part of the exposures. The vehicles were then operated by switching between load and idle with 15-min intervals. During load, the wheel loader buckets were raised, and gas was applied in the upper stage to increase the revolution per minute (rpm) to around 1800–1900 rpm. During idle the engines were kept running without applying extra gas at around 900 rpm. No extra weight was placed in the wheel loader buckets. Both vehicles were run on 100% HVO. The exhaust was extracted from the exhaust pipe of the vehicle, transported in heated tubing and diluted in two steps: first to approximately 1:20–1:30 (heated to 30 °C), and then to a total dilution ratio of 1:160 in the chamber. The setup was previously described by Wierzbicka et al. [15].

#### Filtered air

The filtered air (FA) exposure was obtained by provision of air that passed through a HEPA filter and an active carbon filter. The particle number concentration in the size range 0.02–2.5 µm was on average 71 ± 43 particles cm^−3^, and the volatile organic compound (VOC) concentration < 10 ppb. The FA was used for comparison as a reference exposure.

### Emission characterization

#### Online characterization

The concentrations of PM2.5 mass, NO, and NO_2_ were monitored online during the exposures to keep levels below the pre-determined exposure limits of 150 µg m^−3^, 1 ppm and 2.5 ppm, respectively. The online PM2.5 mass concentration was monitored by an ambient particulate monitor (TEOM series 1400a, Rupprecht & Patashnick Co., N.Y., USA). NO and NO_2_ was measured with a chemiluminescence analyzer (CLD 700 AL, ECO PHYSICS AG, Switzerland). The raw gas emissions of CO and NO_x_ were measured with a flue gas analyzer (Testo 350, Testo AG, Germany) in order to monitor the operation of the vehicles and to ensure exposures below hazardous CO exposure levels (exposure averages were kept below 3 ppm). The CO_2_ concentration in the chamber was monitored with a non-dispersive infrared CO_2_ analyzer (LI-8020, LI-COR, Lincoln, NB, USA) and kept below 1500 ppm. The total VOC concentration (range 10–20,000 ppb) was measured by an online photo-ionization technique (VelociCalc, model 9565-P, probe 986, TSI Inc., U.S.A.).

#### Particle number size distribution and number concentration

The particle number size distributions and concentration in the range 9.8–430 nm (HVO exposures) or 19–914 nm (FA exposures) were measured with a scanning mobility particle sizer (SMPS), consisting of an electrostatic classifier (TSI model 3082) and condensation particle counter (CPC, model 3775, TSI). The aerodynamic size distribution of 0.5–20 µm was monitored with an aerodynamic particle sizer (APS, model 3321, TSI) during the exposures to ensure that the particle number size distributions maxima were captured with the SMPS.

#### Effective density (DMA-APM)

The particle effective density was assessed using an aerosol particle mass analyzer (APM 3600, Kanomax) in combination with a differential mobility analyzer (DMA, TSI Inc., U.S.A.) and a condensation particle counter (CPC, model 3075, TSI Inc., USA) [74]. The effective density was measured at five DMA-selected particle mobility diameters: 50, 70, 100, 150 and 300 nm. Mobility size (d_p_) selection was performed with the DMA. The APM measured the mass distribution of the selected monodisperse aerosol by stepping the voltage for a constant rotating speed. The effective density, ρ_eff,,_ was derived from the arithmetic mean of the measured APM voltage-number distribution and polystyrene latex spheres (PSL, Polymer Microspheres, Duke Scientific Corporation) reference data as described by McMurry et al. [74], shown in Eq. (1):

1 $${\rho }_{eff}={\rho }_{PSL}\frac{{\mathrm{V}}_{APM}}{{\mathrm{V}}_{APM,PSL}}$$

where ρ_PSL_ is the density of the PSL reference particles, V_APM_ is the measured arithmetic mean voltage of the sampled particles for a given mobility diameter and revolutions per minute (rpm), and V_APM,PSL_ is the theoretically calculated arithmetic mean voltage of the PSL reference particles for a given mobility diameter and rpm. The DMA-APM system was calibrated with spherical PSL particles with a known density of 1.05 g cm^−3^.

The effective density of the HVO_PM+NOx_ (soot particles) was fitted assuming a power law function, Eq. (2) [75], where $C{^{\prime}}{{\prime}}$ is a constant and D_fm_ the mass-mobility exponent.

2 $${\rho }_{eff}=C{{\prime}}{{\prime}}{d}_{p}^{{D}_{fm}-3}$$

The mass-mobility relationship was used to extrapolate a power law function for the mobility equivalent particle diameters below 50 nm (up to the inherent material density of soot of 1.8 g cm^−3^) and above 300 nm.

#### Particle mass and surface area size distribution

Mass size distributions were calculated by following Eq. (3), utilizing the particle number size distribution (from the SMPS) and the experimentally determined effective density (ρ_eff_, from the APM) as a function of electrical mobility size (d_p_).

3 $$\mathrm{dM}/{\mathrm{dlogd}}_{\mathrm{p}}=\frac{\uppi {\mathrm{d}}_{\mathrm{p}}^{3}}{6}*{\uprho }_{\mathrm{eff}}({\mathrm{d}}_{\mathrm{p}})*\mathrm{dN}/{\mathrm{dlogd}}_{\mathrm{p}}$$

Lognormal distributions were fitted to the mass size distribution up to 1 µm, and the size-integrated mass concentration was calculated as PM1.

The surface area (SA) distributions were calculated using the model described by Rissler et al. [48], which is based on DMA-APM measurements. From the DMA-APM, the mass of individual agglomerates as a function of mobility particle size can be extracted if the effective density follows the soot power law function (Eq. 2). The surface area of individual agglomerates is then calculated by division of the mass of the agglomerate by the primary particle mass and surface area (SA_pp_ = 6/(ρ_pp_*d_pp_)) [48]. The primary particle size (d_pp_) is obtained from TEM images, and the inherent material density of soot (1.8 g cm^−3^) used for the primary particle density (ρ_pp_). From the surface area of individual agglomerates as a function of mobility particle size, the particle number distribution (from the SMPS) can be converted to a particle surface area distribution. This method accounts for the agglomerated soot structure and is described in more detail by Rissler et al. and Wierzbicka et al. [15, 48].

### Offline characterization

#### Gravimetric analysis

The PM1 mass concentration was determined by gravimetric analysis performed by the Division of Occupational and Environmental Medicine at Örebro University, Örebro, Sweden. The samples were collected during the entire duration of the exposure (180 min) using a PM1 cyclone pre-separator on 37 mm Teflon filters (Zefluor, pore size 1.0 µm) with a flow rate of 5 L min^−1^. The filters were conditioned for 48 h at 50 ± 3% RH and 20 ± 1 °C and weighed before and after collection.

#### Thermal optical carbon analysis and TEM imaging

Samples for the thermal optical analysis of organic carbon (OC) and elemental carbon (EC) were collected on quartz filters (47 mm, Pallflex Tissuequartz) and analyzed with a thermal optical analyzer (DRI Model 2001 OC/EC Carbon Analyzer, Atmoslytic Inc., U.S.A.) using the NIOSH NMAM 5040 diesel exhaust protocol. The limit of detection for EC (LOD) was 0.06 µg C cm^−2^. Two samples were collected in parallel, where one filter collected particle-free air after a Teflon filter (Zefluor, pore size 1.0 µm) which was used to account for gas adsorption artifacts of the filter. Both samples were collected after a PM1 cyclone at a flow rate of 5 L min^−1^ during the entire exposure duration (180 min) and stored refrigerated (+ 6 °C) until analysis.

To analyze the soot particle aggregate structure (morphology and primary particle size), samples were collected with electrostatic precipitation using a nanometer aerosol sampler (model 3089, TSI) on lacey carbon coated Cu-grids and analyzed with a transmission electron microscope (TEM, JEOL 3000F). The TEM was operated at 300 kV and equipped with a Schottky FEG and 2 × 2 k CCD. An overview of the samples was first imaged at 10,000X magnification in order to ensure that the sample was reasonably homogenous. The TEM images of HVO_PM+NOx_ were analyzed for primary particle size determination with the ImageJ software (version 1.52a) [76]. The diameters of the clear primary particles without overlap at the edges of the soot agglomerates were measured in TEM images with a magnification minimum of 25,000X. The diameters of 81 primary particles were measured from 10 agglomerates.

#### PAH analysis

Samples for particulate PAH analysis were collected at a flow rate of 2 L min^−1^ during the entire exposure duration (180 min) on Teflon filters (diameter 37 mm, pore size 2 µm (Teflo, Pall Corporation, Port Washington, N.Y., U.S.A.). These filters were followed by XAD-2 tubes (SKC Inc.) for sampling of gaseous PAHs. The samples were stored at − 18 °C prior to analysis. The samples were analyzed for 33 native and alkylated PAHs (including the 16 U.S. EPA priority PAHs), 17 nitrated, 10 oxygenated PAHs (nitro-PAHs and oxy-PAHs), and 6 dibenzothiophenes (DBTs), as described by Gren et al. 2020 [23]. In brief, prior to extraction two labelled internal standard mixtures containing 16 deuterated U.S. EPA priority PAHs were spiked to the filters and XAD-2 adsorbent, respectively. Samples were extracted with 3 mL dichloromethane, cleaned using silica columns and concentrated to a final volume of approximately 30–40 µL. Target compounds were separated on an Agilent 5975C mass spectrometer (MS) coupled to a 7890A gas chromatograph (GC, Agilent Technologies, Santa Clara, CA, U.S.A.). The MS was operated in selected ion monitoring mode (SIM), and electron impact ionization (EI) was performed for PAHs and alkylated PAHs.

#### Formaldehyde and BTEX analysis

Accredited formaldehyde analysis was performed by the Division of Occupational and Environmental Medicine at Örebro University, Örebro, Sweden. The samples for formaldehyde analysis were collected with a flow rate of 0.2 L min^−1^ during the entire duration of the exposures (180 min) on Sep-Pak DNPH-silica cartridges (Waters). 2,4-dinitrophenylhydrazine (DNPH) formed derivates with aldehydes and were extracted in acetonitrile. The extracted samples were analyzed with liquid chromatography ultraviolet mass spectrometry (LC-UV/MS) at wavelength 360 nm. The samples were stored at − 18 °C prior to analysis.

An accredited analysis of benzene, toluene, ethyl benzene, m + p xylene and o-xylene (BTEX) was performed by the IVL Swedish Environmental Research Institute, Gothenburg, Sweden. n-butyl acetate, n-octane and n-nonane were also analyzed but not included in the total BTEX concentration. The samples were collected on thermal desorption tubes (TENAX TA) and analyzed by a thermal desorption GC–MS method. The sorbent tubes were heated to 250 °C under a helium flow for 5 min. The emitted compounds were refocused with a cold trap (− 30 °C) and then quickly heated to 300 °C in the thermal desorption instrument (Unity2 and Ultra, Markes) and injected into the GC–MS (ThermoFisher Scientific). Target compounds were separated on a non-polar capillary column (TraceGold, TG-1MS, ThermoFisher Scientific) coupled to a mass spectrometer (ISQ LT, ThermoFisher Scientific).

### Model for predicted particle deposition in the respiratory tract

Regional respiratory tract particle deposition fractions from nasal breathing were calculated for the inhaled aerosols with the multiple-path particle dosimetry model (MPPD model version 3.04, [46]). The input parameters are summarized in Table 8. The breathing pattern (respiratory rate and tidal volume) was measured separately. The tidal volume was measured before each exposure and the average value was used for the calculations. The respiratory rate of each volunteer was measured once during 15 min in proximity to one of the exposure sessions with a respiratory inductance plethysmograph (Nox T3 breathing belt, Nox Medical, ResMed) [77].

**Table 8:** The input parameters for the MPPD model used for estimating the respiratory tract particle deposition

MPPD model input data		HVO_PM+NOx_	Diesel [15]
	Model	Yeh/Schum Symmetric	
	Functional residual capacity^a^ (mL) (median, min–max)	3200 (2680–3750)	
	Upper respiratory tract volume (mL)	50	
Particle properties*	Density at MMD (g cm^−3^)	0.84	0.42
	PM1 mass (µg m^−3^) (average ± 1 std. dev.)	93 ± 13	276 ± 56
	Mass Median Diameter (MMD) (µm)	0.108	0.195
	GSD	1.48	1.65
Exposure scenario	Inhalability adjustment	No	
	Acceleration of gravity (m s^−2^)	9.81	
	Body orientation	Upright	
	Respiratory rate^b^ (min^−1^) (median, min–max)	17.1 (13.3–24.9)	
	Tidal volume^c^ (mL) (median, min–max)	875 (440–1500)	
	Inspiratory fraction	0.5	
	Breathing scenario	Nasal	

The aerosol characteristics from a previous diesel exposure study [15] were included and used in the MPPD model to compare the respiratory deposition of HVO and petroleum diesel. The MPPD model’s reference values from ICRP [78] for upper respiratory tract volume and inspiratory fraction were used

^*^Properties from the exposure aerosol characterization. ^a^Calculated by height, age and sex following the guidelines of the European Respiratory Society [79]. ^b^Measured with a respiratory inductance plethysmograph (Nox T3 breathing belt, Nox Medical, ResMed) and analyzed with Noxturnal Software 5.1. ^c^Measured by a forced oscillometry technique with the Tremoflo (THORASYS, Thoracic Medical System Inc., Montreal, Canada)

The predicted inhaled and deposited dose from oral breathing during the HVO_PM+NOx_ exposure was also calculated. This was done with the experimental model reported by Rissler et al. [48] to allow for comparison with a previous exposure study of petroleum diesel exhaust, where this model was used, and was performed with the same exposure setup [15]. The same breathing pattern as in Rissler et al. [48] was used, with a tidal volume om 0.86 L and a respiratory rate of 10.6 min^−1^.

### Medical assessment

#### Nasal patency and pulmonary function measures

Assessment of nasal patency was performed with measurements of peak nasal inspiratory flow (PNIF) using an inspiratory flow meter (In-check, Clement Clarke International Ltd., U.K.) according to the manufacturer’s instructions. Three recordings at each time point (Table 6) were performed and the highest value was used for analysis [80]. The PNIF measurements were compared to the baseline value before the exposure on an individual basis, and a decrease in PNIF indicated an increased nasal obstruction [81].

Lower airway function was assessed with measurements of peak expiratory flow (PEF) measured with a MINI Wright Flow Meter (Clement Clarke International Ltd., U.K.), measuring range of 60–800 L/min. Three recordings at each time point (Table 6) were performed and the highest value was used for analysis [82]. The PEF measurements were compared to the baseline value before the exposure on an individual basis, and a decrease in PEF was used as an indication of lower airway obstruction.

Spirometry was performed with SPIRARE 3 (DIAGNOSTICA, Oslo, Norway) according to the European Respiratory Society Guidelines [82]. Forced vital capacity (FVC), forced expiratory flow in the first second (FEV_1_), FEV1/FVC, and z-scores were obtained and compared according to the reference of the Global Lung Initiative [83].

Measures of oscillometry (resistance [R], reactance [X]) were obtained in a frequency range of 5–19 Hz by the forced oscillometric technique (FOT), with a Tremoflo (THORASYS, Thoracic Medical System Inc., Canada) according to the manufacturer’s instructions [49]. The resistance at 5 and 19 Hz (R_5_, R_19_), reactance at 5 Hz, area under the reactance curve from 5 to 19 Hz (A_X_), and the resonant frequency (F_res_) before all exposures were averaged and summarized for all volunteers in Additional file 1: G. The individual oscillograms were evaluated by a trained physician. Some volunteers had FOT values deviating from the normal range [49, 50] but with normal spirometry measures, thus the volunteers were further categorized into a “typical” and “atypical” group based on their oscillometric measurements. The criteria were as follows: R_5–19_ ≥ 0.8 cmH_2_O s L^−1^, X_5_ ≤ − 1.8 cmH_2_O s L^−1^, and A_x_ ≥ 14 cmH_2_O L^−1^ (Additional file 1: G). These criteria were based on characteristics of healthy and asthmatic subjects as published by Eddy et al. [50]. The atypical group showed a higher proportion of having a history of symptoms and atopy (80% vs. 54%, p < 0.05). They were hypothesized to be more sensitive and to have a different lung reaction than the typical group, which was explored further in the analyses.

#### Self-rated symptoms

Similar to a previous study [6], self-rated symptoms of eye irritation, nose irritation (including runny nose and nasal congestion), throat irritation and chest tightness/breathlessness were rated by the volunteers themselves on a visual analog scale (VAS) (range 0 to 100 mm) before exposure, and at 35, 95 and 155 min into the exposure during each exposure session (Table 6).

### Statistical analysis

For each self-rated symptom (eye, nose, throat, and chest), when a volunteer gave a higher score than before the exposure at any time during the exposure, this person was recoded as “reported symptoms”, otherwise, “no reported symptoms”. A person was then recoded as “reported any symptom” if he/she reported any of the four symptoms during the exposure. The calculation was performed for each exposure scenario separately. Descriptive analysis was used to count the number of persons and corresponding proportion of persons with reported symptoms during exposure for each exposure scenario. An ${\chi }^{2}$-test was used to investigate the difference in proportion between the given exposure scenarios (each of the two exposure scenarios) in comparison to FA exposure when applicable.

For PNIF and PEF measurements that were performed three times during each exposure, absolute changes from before exposure were calculated on the individual level for each exposure scenario at each time point. Linear mixed models were used to analyze the average changes in the selected outcomes at given exposure scenarios versus changes at FA exposure. Subject ID, the exposure scenarios and time points [1–4] were used to identify repeated measurements with repeated covariance type as Autoregressive. All models included a random slope, allowing the effects of exposures to vary for each individual. Since absolute changes were used in the model, each individual had an intercept as 0 for each exposure scenario and therefore no random intercept was considered. The models included exposure scenarios, time points and exposure order (e.g., first or second time in the chamber) as the fixed factors, the latter was included due to imbalanced order and a learning effect on the measurement performance might have occurred.

For spirometry and FOT measurements, which were only performed before and after exposure, the Wilcoxon signed-rank test was used to compare the differences between before and after exposure at each exposure scenario.

Additionally, interaction terms between exposure scenarios and typical/atypical groups were tested for PNIF, PEF and with the FOT in the linear mixed models described above, to see if the atypical group with FOT measurements outside the normal range (Additional file 1: G) showed different exposure-related changes in nasal patency and pulmonary function. If the interaction terms had a p-value < 0.05, further stratified analyses were performed in each group for selected outcomes.

All statistical analyses were performed using IBM SPSS Statistics 26. For all tests, p-values < 0.1 were considered as weak indications, while P values < 0.05 were generally accepted as significant.

## Supplementary Information

**Additional file 1:** Supplemental tables and figures.

## Data Availability

The datasets used and/or analyzed during the current study are available from the corresponding author on reasonable request.

## Abbreviations

APM: Aerosol particle mass analyzer
BTEX: Benzene, toluene, ethyl benzene, m + p xylene, o-xylene
CI: Confidence interval
DMA: Differential mobility analyzer
DPF: Diesel particle filter
DOC: Diesel oxidation catalyst
EC: Elemental carbon
FA: Filtered air
FEV: Forced expiratory volume
FOT: Forced oscillation technique
FVC: Forced vital capacity
HVO: Hydrotreated vegetable oil
MMD: Mass median diameter
MPPD: Multiple-path particle dosimetry model
NO: Nitrogen monoxide
NO_2_: Nitrogen dioxide
NO_x_: Nitrogen oxides
OELs: Occupational exposure limits
PAHs: Polycyclic aromatic hydrocarbons
PEF: Peak expiratory flow
PM: Particulate matter
PN: Particle number
PNIF: Peak nasal inspiratory flow
SA: Surface area
TEM: Transmission electron microscope
VAS: Visual analog scale
VOC: Volatile organic compound
