# Supplementary material for: Lung function and self-rated symptoms in healthy volunteers after exposure to hydrotreated vegetable oil (HVO) exhaust with and without particles
Source: Part Fibre Toxicol. 2022 Jan 24;19:9. doi: 10.1186/s12989-021-00446-7 (PMC8785558; doi:10.1186/s12989-021-00446-7)
Supplement: Supplementary file 1 — Additional file 1: Supplemental tables and figures. [file 12989_2021_446_MOESM1_ESM.docx]

**Additional file**

Lung function and self-rated symptoms in healthy volunteers after exposure to hydrotreated vegetable oil (HVO) exhaust with and without particles

Louise Gren^1,2^, Katrin Dierschke^3^, Fredrik Mattsson^1^, Eva Assarsson^3^, Annette M. Krais^3^, Monica Kåredal^2,3^, Karin Lovén^1^, Jakob Löndahl^1,2^, Joakim Pagels^1,2^, Bo Strandberg^3^, Martin Tunér^4^, Yiyi Xu^5^, Per Wollmer^6^, Maria Albin^3,7^, Jörn Nielsen^3^, Anders Gudmundsson^1,2^, Aneta Wierzbicka^1,8*^

^1^ Ergonomics and Aerosol Technology, Lund University, Lund, SE-221 00, Sweden

^2^ Lund University, NanoLund, SE-221 00 Lund, Sweden

^3^ Division of Occupational and Environmental Medicine, Lund University, Lund, SE-223 63, Sweden

^4^ Division of Combustion Engines, Lund University, Lund, SE-221 00, Sweden

^5^ School of Public Health and Community Medicine, Institute of Medicine, University of Gothenburg, Sweden

^6^ Department of Translational Medicine, Lund University, Sweden

^7^ Unit of Occupational Medicine, Institute of Environmental Medicine, Karolinska Institute, Stockholm, Sweden

^8^ Centre for Healthy Indoor Environments, Lund University, 221 00 Lund, Sweden

*Corresponding author: [aneta.wierzbicka@design.lth.se](mailto:aneta.wierzbicka@design.lth.se)

**A. Detailed PAH analysis**

**Table 1.** Average concentrations (ng m^-3^) of the particle phase PAHs measured during the 3 h exposures.

|  | **HVO_PM+Nox_** | | **HVO_Nox_** | | **FA** | | **LOD** |
| --- | --- | --- | --- | --- | --- | --- | --- |
| **Particle phase (ng/m^3^)** | Average | Std. Dev | Average | Std. Dev | Average | Std. Dev |  |
| Number of samples (n) | 8 | | 6 | | 2^a^ | |  |
| **Native and alkylated PAHs** |  |  |  |  |  |  |  |
| naphthalene | 2.3 | 1.7 | 0.74 | -* | <LOD |  | <0.58 |
| 2-methylnaphthalene | 0.61 | 0.16 | 0.77 | -* | <LOD |  | <0.39 |
| 1-methylnaphthalene | 0.92 | 0.17 | <LOD |  | <LOD |  | <0.69 |
| biphenyl | <LOD |  | <LOD |  | <LOD |  | <0.40 |
| 2,3-dimethylnaphthalene | 0.30 | -* | <LOD |  | <LOD |  | <0.27 |
| acenaphthylene | <LOD |  | <LOD |  | <LOD |  | <0.058 |
| acenaphthene | 0.23 | 0.098 | <LOD |  | <LOD |  | <0.12 |
| 2,3,5-trimethylnaphthalene | <LOD |  | <LOD |  | <LOD |  | <0.47 |
| fluorene | 0.30 | 0.23 | 0.20 | -* | <LOD |  | <0.023 |
| 1-methylfluorene | <LOD |  | <LOD |  | <LOD |  | <0.11 |
| phenanthrene | 2.6 | 0.35 | <LOD |  | <LOD |  | <0.17 |
| anthracene | 0.52 | 0.18 | <LOD |  | <LOD |  | <0.013 |
| 2-methylphenanthrene | 0.14 | 0.056 | <LOD |  | <LOD |  | <0.021 |
| 3-methylphenanthrene | 0.25 | 0.067 | <LOD |  | <LOD |  | <0.0085 |
| 1-methylphenanthrene | 0.33 | 0.16 | <LOD |  | <LOD |  | <0.0077 |
| 1-methylanthracene | 0.21 | 0.063 | <LOD |  | <LOD |  | <0.027 |
| 2-phenylnaphthalene | 0.48 | 0.10 | <LOD |  | <LOD |  | <0.037 |
| fluoranthene | 11 | 1.2 | <LOD |  | <LOD |  | <0.040 |
| pyrene | 13 | 1.4 | <LOD |  | <LOD |  | <0.0077 |
| 1-methylfluoranthene | 0.29 | 0.027 | <LOD |  | <LOD |  | <0.0031 |
| retene | 0.12 | 0.074 | 0.024 | -* | <LOD |  | <0.0038 |
| 1-methylpyrene | 0.24 | 0.017 | <LOD |  | <LOD |  | <0.0062 |
| benzo(a)anthracene | 0.086 | 0.047 | <LOD |  | <LOD |  | <0.0065 |
| chrysene | 1.0 | 0.10 | <LOD |  | <LOD |  | <0.024 |
| 2-methylchrysene | 0.031 | -* | <LOD |  | <LOD |  | <0.029 |
| benzo(b)fluoranthene | 1.1 | 0.12 | 0.082 | -* | <LOD |  | <0.077 |
| benzo(k)fluoranthene | 0.21 | 0.068 | 0.047 | -* | <LOD |  | <0.016 |
| benzo(a)pyrene | 0.52 | 0.090 | 0.034 | -* | 0.070 | -* | <0.012 |
| perylene | <LOD |  | <LOD |  | <LOD |  | <0.015 |
| indeno(1,2,3-c,d)pyrene | 0.50 | 0.1205 | <LOD |  | 0.12 | -* | <0.0044 |
| dibenzo(a,h)anthracene | 0.048 | 0.0185 | <LOD |  | <LOD |  | <0.012 |
| benzo(g,h,i)perylene | 1.1 | 0.1409 | <LOD |  | 0.10 | -* | <0.0041 |
| coronene | <LOD |  | <LOD |  | <LOD |  | <0.0040 |
| **Sum native and alkylated PAHs** | **38** | **3.5** | **0.32** | **0.71** | **0.15** | **0.15** |  |
|  |  |  |  |  |  |  |  |
| **Dibenzothiophenes** |  |  |  |  |  |  |  |
| dibenzothiophene | 0.046 | 0.013 | <LOD |  | <LOD |  | <0.027 |
| 2-methyldibenzothiophene | 0.064 | 0.019 | <LOD |  | <LOD |  | <0.0090 |
| 1-methyldibenzothiophene | <LOD | <LOD | <LOD |  | <LOD |  | <0.077 |
| 4-methyldibenzothiophene | <LOD | <LOD | <LOD |  | <LOD |  | <0.0076 |
| 2,8-dimethyldibenzothiophene | 0.24 | 0.10 | 0.015 | -* | <LOD |  | <0.014 |
| 2,4,7-trimethyldibenzothiophene | 0.14 | 0.028 | 0.010 | -* | 0.016 | -* | <0.0047 |
| **Sum DBTs** | **0.50** | **0.12** | **0.025** | **0.01** | **0.01** | **0.01** |  |
|  |  |  |  |  |  |  |  |
| **Nitro-PAHs** |  |  |  |  |  |  |  |
| 1-Nitronaphthalene | 0.011 | -* | <LOD |  | <LOD |  | <0.0032 |
| 2-Nitronapthalene | 0.0096 | 0.011 | <LOD |  | <LOD |  | <0.0013 |
| 5-nitro acenapthalene | 0.20 | 0.18 | <LOD |  | <LOD |  | <0.018 |
| 2-Nitrofluorene | 0.028 | 0.017 | <LOD |  | <LOD |  | <0.010 |
| 9-Nitroanthracene | <LOD | <LOD | <LOD |  | <LOD |  | <0.0029 |
| 9-Nitrophenanthrene | 0.012 | -* | <LOD |  | <LOD |  | <0.0064 |
| 4-Nitropyrene | <LOD | <LOD | <LOD |  | <LOD |  | <0.0064 |
| 3-Nitrofluoranthene | 0.054 | 0.011 | <LOD |  | <LOD |  | <0.0040 |
| 1-Nitropyrene | 0.13 | 0.034 | <LOD |  | <LOD |  | <0.040 |
| 2-Nitropyrene | <LOD | <LOD | <LOD |  | <LOD |  | <0.0079 |
| 7-Nitrobenz[a]anthracene | 0.18 | 0.064 | <LOD |  | <LOD |  | <0.043 |
| 6-Nitrochrysene | 0.015 | -* | <LOD |  | <LOD |  | <0.023 |
| 3-Nitrobenzanthrone | <LOD | <LOD | <LOD |  | <LOD |  | <0.035 |
| 1,3-Dinitropyrene | <LOD | <LOD | <LOD |  | <LOD |  | <0.048 |
| 1,6-Dinitropyrene | <LOD | <LOD | <LOD |  | <LOD |  | <0.027 |
| 1,8-Dinitropyrene | <LOD | <LOD | <LOD |  | <LOD |  | <0.0080 |
| 6-Nitrobenzo[a]pyrene | <LOD | <LOD | <LOD |  | <LOD |  | <0.037 |
| **Sum Nitro-PAHs** | **0.64** | **0.27** | **0.00** |  | **0.00** |  |  |
|  |  |  |  |  |  |  |  |
| **Oxy-PAHs** |  |  |  |  |  |  |  |
| Napthalene-1-aldehyde | 0.38 | 0.19 | <LOD |  | <LOD |  | <0.082 |
| 2-Naphthaldehyde | 0.11 | 0.023 | <LOD |  | <LOD |  | <0.069 |
| p-Fluorenone | 0.41 | 0.18 | <LOD |  | <LOD |  | <0.048 |
| 9,10 Anthraquinone | 0.59 | 0.21 | <LOD |  | <LOD |  | <0.032 |
| 1,4 Anthraquinone | 0.019 | 0.0070 | <LOD |  | <LOD |  | <0.0065 |
| Phenanthrene-9-aldehyde | 0.51 | 0.077 | <LOD |  | <LOD |  | <0.0088 |
| Benzo[a]fluorene | 0.23 | 0.070 | <LOD |  | <LOD |  | <0.0099 |
| Benzo[b]fluorene | 0.090 | 0.12 | <LOD |  | <LOD |  | <0.0058 |
| Benzanthrone | 0.72 | 0.23 | <LOD |  | 0.050 | <LOD* | <0.015 |
| Benz[a]anthracene-7,12-dione | 0.15 | 0.030 | <LOD |  | 0.098 | <LOD* | <0.070 |
| **Sum Oxy-PAHs** | **3.2** | **0.70** | **0.00** |  | **0.07** | **0.07** |  |
|  |  |  |  |  |  |  |  |
| **Sum total PAHs** | **43** | **4.9** | **0.32** | **0.79** | **0.23** | **0.32** |  |
| ^a^One sample was excluded due to contamination during sampling.  *Only one (1) measurement >LOD, no std. dev. available. | | | | | | | |

**Table 2.** Average concentrations (ng m^-3^) of the gas phase PAHs measured during the 3 h exposures.

|  | **HVO_PM+Nox_** | | **HVO_Nox_** | | **FA** | | **LOD** |
| --- | --- | --- | --- | --- | --- | --- | --- |
| **Gas phase (ng/m^3^)** | Average | Std. Dev | Average | Std. Dev | Average | Std. Dev |  |
| Number of samples (n) | 8 | | 6 | | 2^a^ | |  |
| **Native and alkylated PAHs** |  |  |  |  |  |  |  |
| naphthalene | 500 | 41 | 31. | 8.4 | 49 | 23 | <0.58 |
| 2-methylnaphthalene | 32 | 6.4 | 14 | 3.0 | 15 | 0.66 | <0.39 |
| 1-methylnaphthalene | 24 | 4.1 | 6.6 | 1.4 | 7.5 | 0.85 | <0.69 |
| biphenyl | 20 | 5.7 | 4.3 | 1.1 | 5.8 | 1.1 | <0.40 |
| 2,3-dimethylnaphthalene | 2.1 | -* | 2.4 | 1.0 | 1.3 | 0.95 | <0.27 |
| acenaphthylene | 16 | 2.4 | 1.2 | 0.48 | 1.7 | 0.46 | <0.058 |
| acenaphthene | 220 | 46 | 21 | 11 | 14 | 3.8 | <0.12 |
| 2,3,5-trimethylnaphthalene | 1.3 | 0.46 | 1.1 | 0.58 | <LOD |  | <0.47 |
| fluorene | 8.2 | 0.97 | 7.9 | 0.57 | 10 | 1.3 | <0.023 |
| 1-methylfluorene | 0.76 | 0.20 | 0.29 | 0.12 | 0.52 | 0.093 | <0.11 |
| phenanthrene | 8.7 | 0.96 | 5.5 | 1.2 | 7.3 | 0.56 | <0.17 |
| anthracene | 0.39 | 0.074 | 0.23 | 0.057 | 0.42 | 0.042 | <0.013 |
| 2-methylphenanthrene | 0.17 | 0.072 | 0.19 | 0.037 | 0.15 | -* | <0.021 |
| 3-methylphenanthrene | 0.15 | 0.071 | 0.17 | 0.11 | 0.078 | 0.062 | <0.0085 |
| 1-methylphenanthrene | 0.11 | 0.057 | 0.10 | 0.038 | 0.24 | 0.19 | <0.0077 |
| 1-methylanthracene | 0.063 | 0.025 | 0.063 | 0.0096 | 0.10 | -* | <0.027 |
| 2-phenylnaphthalene | 0.041 | -* | <LOD |  | 0.17 | -* | <0.037 |
| fluoranthene | 0.10 | 0.040 | 0.12 | 0.016 | 0.17 | 0.066 | <0.040 |
| pyrene | 0.035 | - * | <LOD |  | <LOD |  | <0.0077 |
| 1-methylfluoranthene | <LOD |  | <LOD |  | <LOD |  | <0.0031 |
| retene | 0.028 | 0.015 | 0.033 | -* | 0.017 | -* | <0.0038 |
| 1-methylpyrene | <LOD |  | <LOD |  | <LOD |  | <0.0062 |
| benzo(a)anthracene | <LOD |  | <LOD |  | <LOD |  | <0.0065 |
| chrysene | <LOD |  | <LOD |  | <LOD |  | <0.024 |
| 2-methylchrysene | <LOD |  | <LOD |  | <LOD |  | <0.029 |
| benzo(b)fluoranthene | <LOD |  | <LOD |  | <LOD |  | <0.077 |
| benzo(k)fluoranthene | <LOD |  | <LOD |  | <LOD |  | <0.016 |
| benzo(a)pyrene | <LOD |  | <LOD |  | <LOD |  | <0.012 |
| perylene | <LOD |  | <LOD |  | <LOD |  | <0.015 |
| indeno(1,2,3-c,d)pyrene | <LOD |  | <LOD |  | <LOD |  | <0.0044 |
| dibenzo(a,h)anthracene | <LOD |  | <LOD |  | <LOD |  | <0.012 |
| benzo(g,h,i)perylene | <LOD |  | <LOD |  | <LOD |  | <0.0041 |
| coronene | <LOD |  | <LOD |  | <LOD |  | <0.0040 |
| **Sum native and alkylated PAHs** | **840** | **49** | **95** | **19** | **110** | **28** |  |
|  |  |  |  |  |  |  |  |
| **Dibenzothiophenes** |  |  |  |  |  |  |  |
| dibenzothiophene | 0.92 | 0.35 | 0.47 | 0.23 | 0.77 | 0.10 | <0.027 |
| 2-methyldibenzothiophene | 0.26 | 0.11 | 0.16 | 0.064 | 0.24 | 0.11 | <0.0090 |
| 1-methyldibenzothiophene | 0.44 | 0.21 | 0.19 | 0.076 | 0.37 | 0.15 | <0.077 |
| 4-methyldibenzothiophene | 0.031 | 0.013 | 0.014 | 0.0040 | 0.018 | 0.0037 | <0.0076 |
| 2,8-dimethyldibenzothiophene | 0.092 | 0.035 | 0.059 | 0.026 | 0.12 | 0.030 | <0.014 |
| 2,4,7-trimethyldibenzothiophene | 0.0092 | -* | 0.0091 | -* | 0.012 | 0.0010 | <0.0047 |
| **Sum DBTs** | **1.8** | **0.67** | **0.67** | **0.43** | **1.5** | **0.20** |  |
|  |  |  |  |  |  |  |  |
| **Nitro-PAHs** |  |  |  |  |  |  |  |
| 1-Nitronaphthalene | 0.029 | 0.0047 | 0.024 | 0.016 | 0.0075 | 0.0009 | <0.0032 |
| 2-Nitronapthalene | 0.049 | 0.021 | 0.015 | 0.0095 | 0.018 | 0.017 | <0.0013 |
| 5-nitro acenapthalene | <LOD |  | <LOD |  | <LOD |  | <0.018 |
| 2-Nitrofluorene | 0.013 | -* | <LOD |  | <LOD |  | <0.010 |
| 9-Nitroanthracene | <LOD |  | <LOD |  | <LOD |  | <0.0029 |
| 9-Nitrophenanthrene | <LOD |  | <LOD |  | <LOD |  | <0.0064 |
| 4-Nitropyrene | <LOD |  | <LOD |  | <LOD |  | <0.0064 |
| 3-Nitrofluoranthene | <LOD |  | <LOD |  | <LOD |  | <0.0040 |
| 1-Nitropyrene | <LOD |  | <LOD |  | <LOD |  | <0.040 |
| 2-Nitropyrene | <LOD |  | <LOD |  | <LOD |  | <0.0079 |
| 7-Nitrobenz[a]anthracene | <LOD |  | <LOD |  | <LOD |  | <0.043 |
| 6-Nitrochrysene | <LOD |  | <LOD |  | <LOD |  | <0.023 |
| 3-Nitrobenzanthrone | <LOD |  | <LOD |  | <LOD |  | <0.035 |
| 1,3-Dinitropyrene | <LOD |  | <LOD |  | <LOD |  | <0.048 |
| 1,6-Dinitropyrene | <LOD |  | <LOD |  | <LOD |  | <0.027 |
| 1,8-Dinitropyrene | <LOD |  | <LOD |  | <LOD |  | <0.0080 |
| 6-Nitrobenzo[a]pyrene | <LOD |  | <LOD |  | <LOD |  | <0.037 |
| **Sum Nitro-PAHs** | **0.09** | **0.03** | **0.04** | **0.02** | **0.03** | **0.02** |  |
|  |  |  |  |  |  |  |  |
| **Oxy-PAHs** |  |  |  |  |  |  |  |
| Napthalene-1-aldehyde | 4.8 | 1.1 | 0.61 | 0.37 | 0.58 | 0.32 | <0.082 |
| 2-Naphthaldehyde | 2.0 | 0.32 | 0.36 | 0.25 | 0.24 | 0.12 | <0.069 |
| p-Fluorenone | 1.2 | 0.18 | 0.88 | 0.44 | 0.33 | 0.096 | <0.048 |
| 9,10 Anthraquinone | 0.10 | 0.049 | 0.17 | 0.027 | 0.18 | - * | <0.032 |
| 1,4 Anthraquinone | 0.015 | -* | <LOD |  | <LOD |  | <0.0065 |
| Phenanthrene-9-aldehyde | <LOD |  | 0.011 | 0.0025 | 0.011 | -* | <0.0088 |
| Benzo[a]fluorene | <LOD |  | 0.013 | - * | <LOD |  | <0.0099 |
| Benzo[b]fluorene | <LOD |  | <LOD |  | <LOD |  | <0.0058 |
| Benzanthrone | <LOD |  | <LOD |  | <LOD |  | <0.015 |
| Benz[a]anthracene-7,12-dione | <LOD |  | <LOD |  | <LOD |  | <0.070 |
| **Sum Oxy-PAHs** | **8.2** | **1.4** | **1.9** | **1.1** | **1.2** | **0.62** |  |
|  |  |  |  |  |  |  |  |
| **Sum total PAHs** | **850** | **51** | **98** | **20** | **120** | **29** |  |
| ^a^ One sample was excluded due to contamination during sampling.  *Only one (1) measurement >LOD, no std. dev. Available. | | | | | | | |

**B. Detailed BTEX analysis**

**Table.** The average concentration of the VOCs (µg m^-3^) categorized as BTEX during the 3 h exposures.

|  | **HVO_PM+Nox_** | **HVO_Nox_** | **FA** |
| --- | --- | --- | --- |
| Number of samples (n) | 6 | 5 | 2 |
| **Compound (µg m^-3^)** | Average ± Std. Dev. | Average ± Std. Dev. | Average ± Std. Dev. |
| benzene | 5.83 ± 2.35 | 0.64 ± 0.19 | 0.16 ± 0.04 |
| toluene | 1.23 ± 0.15 | 0.38 ± 0.09 | 0.33 ± 0.06 |
| n-butyl acetate | 0.26 ± 0.16 | 0.54 ± 0.63 | 0.38 ± 0.26 |
| n-octane | 1.79 ± 0.41 | 0.78 ± 0.11 | 0.16 ± 0.02 |
| ethyl benzene | 0.23 ± 0.02 | 0.14 ± 0.03 | 0.24 ± 0.03 |
| m+p-xylene | 0.42 ± 0.06 | 0.37 ± 0.05 | 0.39 ± 0 |
| o-xylene | 0.15 ± 0.02 | 0.13 ± 0.02 | 0.15 ± 0.02 |
| n-nonane | 1.7 ± 0.21 | 0.56 ± 0.05 | 0.13 ± 0.01 |

**C. Lung deposition**


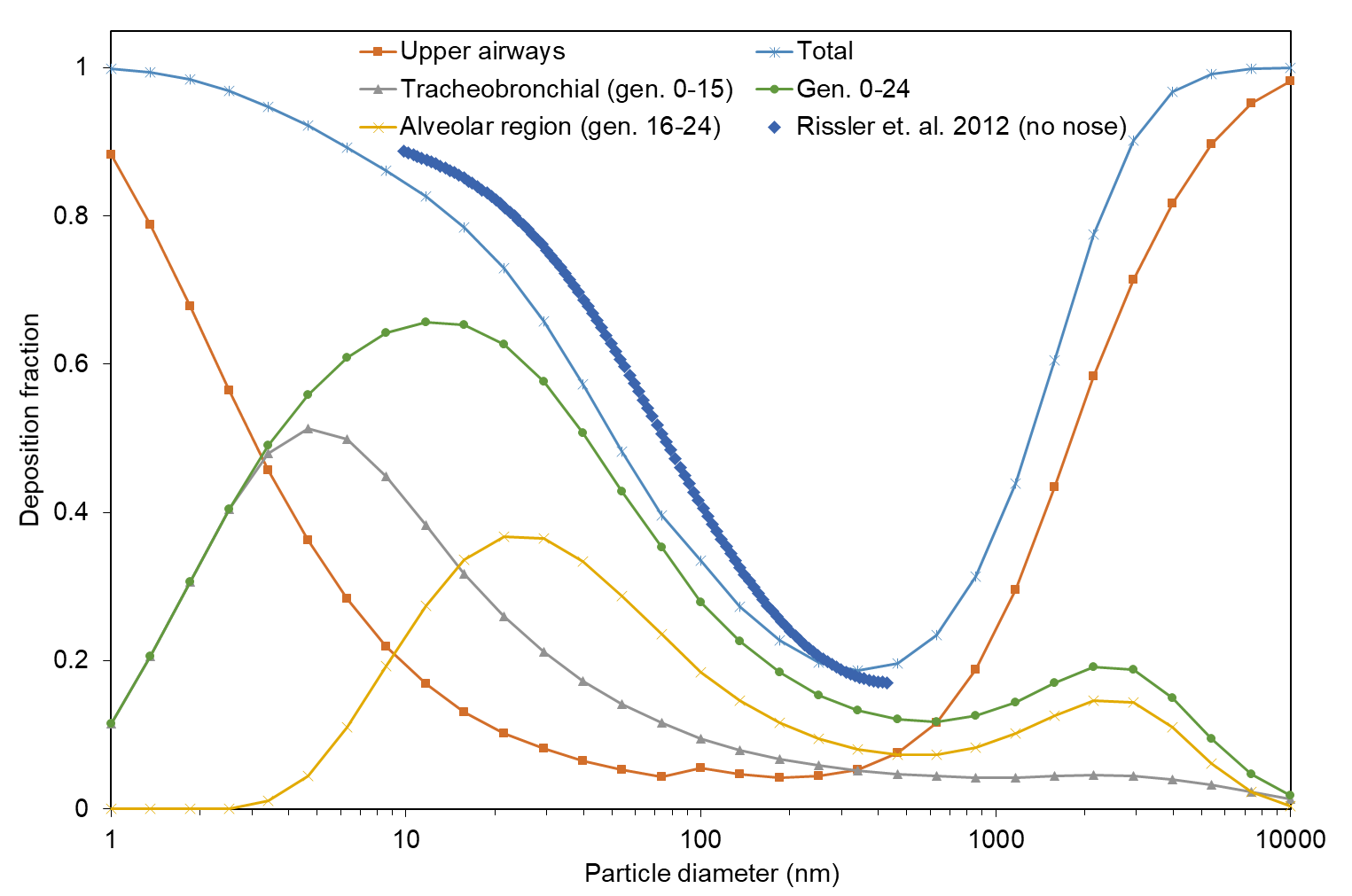


**Figure.** The lung deposition fraction depending on particle diameter, from the nasal breathing employed in the MPPD model (upper airways, tracheobronchial and alveolar region, and total deposition) (3) and from oral breathing in Rissler et al. (4).

**D. Self-reported symptoms score**

**Table**. Self-reported score symptoms (on a 100 mm VAS scale) in different exposures at different timepoints.

|  |  |  | **Eye symptoms**  (Itching, running and/or sore eyes) | | | | | **Nose symptoms**  (Running nose) | | | | | **Nose symptoms**  (Congestion) | | | | | **Throat symptoms**  (Sore/dry/irritated throat) | | | | | **Chest symptoms**  (Chest tightness/breathlessness) | | | | |
| --- | --- | --- | --- | --- | --- | --- | --- | --- | --- | --- | --- | --- | --- | --- | --- | --- | --- | --- | --- | --- | --- | --- | --- | --- | --- | --- | --- |
| **Exposure** | **Time point** | Count (N) | Mean | 25% | Median | 75% | Max | Mean | 25% | Median | 75% | Max | Mean | 25% | Median | 75% | Max | Mean | 25% | Median | 75% | Max | Mean | 25% | Median | 75% | Max |
| FA | Before | 17 | 0 | 0 | 0 | 0 | 1 | 0 | 0 | 0 | 0 | 2 | 0 | 0 | 0 | 0 | 4 | 1 | 0 | 0 | 0 | 7 | 0 | 0 | 0 | 0 | 2 |
|  | 45 min | 18 | 0 | 0 | 0 | 0 | 0 | 0 | 0 | 0 | 0 | 0 | 0 | 0 | 0 | 0 | 0 | 0 | 0 | 0 | 0 | 5 | 0 | 0 | 0 | 0 | 0 |
|  | 105 min | 18 | 0 | 0 | 0 | 0 | 3 | 0 | 0 | 0 | 0 | 0 | 1 | 0 | 0 | 0 | 12 | 0 | 0 | 0 | 0 | 3 | 0 | 0 | 0 | 0 | 0 |
|  | 155 min | 18 | 1 | 0 | 0 | 0 | 5 | 0 | 0 | 0 | 0 | 0 | 0 | 0 | 0 | 0 | 0 | 0 | 0 | 0 | 0 | 5 | 0 | 0 | 0 | 0 | 0 |
| HVO_NOx_ | Before | 19 | 0 | 0 | 0 | 0 | 7 | 0 | 0 | 0 | 0 | 0 | 0 | 0 | 0 | 0 | 9 | 1 | 0 | 0 | 0 | 9 | 0 | 0 | 0 | 0 | 8 |
|  | 45 min | 19 | 1 | 0 | 0 | 0 | 8 | 0 | 0 | 0 | 0 | 0 | 0 | 0 | 0 | 0 | 0 | 2 | 0 | 0 | 0 | 17 | 0 | 0 | 0 | 0 | 0 |
|  | 105 min | 19 | 2 | 0 | 0 | 4 | 19 | 0 | 0 | 0 | 0 | 4 | 0 | 0 | 0 | 0 | 0 | 3 | 0 | 0 | 3 | 40 | 0 | 0 | 0 | 0 | 2 |
|  | 155 min | 19 | 2 | 0 | 0 | 0 | 25 | 1 | 0 | 0 | 0 | 12 | 0 | 0 | 0 | 0 | 0 | 2 | 0 | 0 | 4 | 21 | 0 | 0 | 0 | 0 | 0 |
| HVO_PM+Nox_ | Before | 18 | 0 | 0 | 0 | 0 | 0 | 1 | 0 | 0 | 0 | 7 | 0 | 0 | 0 | 0 | 0 | 1 | 0 | 0 | 0 | 13 | 0 | 0 | 0 | 0 | 0 |
|  | 45 min | 18 | 5 | 0 | 0 | 4 | 47 | 0 | 0 | 0 | 0 | 3 | 1 | 0 | 0 | 0 | 10 | 2 | 0 | 0 | 0 | 19 | 1 | 0 | 0 | 0 | 9 |
|  | 105 min | 18 | 6 | 0 | 0 | 12 | 29 | 0 | 0 | 0 | 0 | 3 | 2 | 0 | 0 | 0 | 13 | 4 | 0 | 0 | 3 | 29 | 1 | 0 | 0 | 0 | 7 |
|  | 155 min | 18 | 4 | 0 | 0 | 10 | 24 | 1 | 0 | 0 | 0 | 6 | 3 | 0 | 0 | 0 | 29 | 5 | 0 | 0 | 4 | 34 | 1 | 0 | 0 | 0 | 7 |

**E. Absolute PNIF and PEF values**

**Table**. The absolute PNIF and PEF values.

|  | Timepoint  (min into exposure) | **PNIF (L min^-1^)** | | | | **PEF (L min^-1^)** | | | |
| --- | --- | --- | --- | --- | --- | --- | --- | --- | --- |
|  |  | Average  ± Std. Dev | Median | Minimum | Maximum | Average  ± Std. Dev | Median | Minimum | Maximum |
| FA | Before | 123 ± 35 | 110 | 80 | 173 | 509 ± 101 | 515 | 340 | 690 |
|  | 55 min | 136 ± 37 | 140 | 80 | 177 | 509 ± 105 | 540 | 330 | 650 |
|  | 115 min | 142 ± 37 | 153 | 70 | 173 | 510 ± 111 | 525 | 300 | 670 |
|  | 175 min | 130 ± 26 | 135 | 70 | 157 | 509 ± 102 | 540 | 320 | 650 |
| HVO_NOx_ | Before | 146 ± 39 | 150 | 80 | 230 | 517 ± 89 | 540 | 370 | 650 |
|  | 55 min | 142 ± 44 | 150 | 70 | 230 | 519 ± 91 | 540 | 360 | 660 |
|  | 115 min | 143 ± 45 | 150 | 60 | 230 | 525 ± 90 | 530 | 370 | 650 |
|  | 175 min | 139 ± 37 | 140 | 80 | 200 | 521 ± 89 | 490 | 350 | 650 |
| HVO_PM+NOx_ | Before | 128 ± 37 | 130 | 70 | 200 | 503 ± 95 | 540 | 370 | 650 |
|  | 55 min | 130 ± 39 | 123 | 70 | 210 | 502 ± 93 | 540 | 360 | 660 |
|  | 115 min | 129 ± 35 | 140 | 75 | 190 | 506 ± 97 | 530 | 370 | 650 |
|  | 175 min | 131 ± 30 | 140 | 90 | 210 | 502 ± 100 | 490 | 350 | 650 |

**F. Spirometry**

**Table 1.** The FVC and FEV_1_ in absolute values (L) as Z-scores and in % of predicted values before and after the exposures. The median difference was analyzed using the paired non-parametric test (Wilcoxon signed-rank test) for each exposure scenario.

|  |  | Before exposure | | | | | | After exposure | | | | | | | | | | | Paired test | |
| --- | --- | --- | --- | --- | --- | --- | --- | --- | --- | --- | --- | --- | --- | --- | --- | --- | --- | --- | --- | --- |
|  |  |  |  |  | Percentiles | | |  | | |  | |  | | Percentiles | | | |  | |
|  |  | Avg. | Std. Dev. | Median | 25th | 75th | | Avg. | | | Std. Dev. | | Median | | 25th | | 75th | | p-values | |
| FEV_1_ (L) | Filtered air | 3.38 | 0.60 | 3.25 | 3.19 | | 3.61 | | 3.40 | 0.55 | | 3.35 | | 3.14 | | 3.62 | | 0.331 | |  |
|  | HVO_NOx_ | 3.36 | 0.54 | 3.32 | 3.15 | | 3.55 | | 3.37 | 0.58 | | 3.33 | | 3.07 | | 3.55 | | 0.409 | |  |
|  | HVO_PM+NOx_ | 3.46 | 0.60 | 3.27 | 3.20 | | 3.71 | | 3.47 | 0.62 | | 3.29 | | 3.23 | | 3.79 | | 0.602 | |  |
| FVC (L) | Filtered air | 4.24 | 0.76 | 3.84 | 3.77 | | 4.42 | | 4.26 | 0.75 | | 4.01 | | 3.67 | | 4.46 | | 0.477 | |  |
|  | HVO_NOx_ | 4.28 | 0.73 | 3.92 | 3.69 | | 4.75 | | 4.26 | 0.79 | | 3.92 | | 3.66 | | 4.94 | | 0.408 | |  |
|  | HVO_PM+NOx_ | 4.22 | 0.70 | 3.97 | 3.76 | | 4.36 | | 4.21 | 0.73 | | 4.01 | | 3.69 | | 4.40 | | 0.687 | |  |
| FEV_1_/FVC | Filtered air | 0.80 | 0.06 | 0.81 | 0.74 | | 0.85 | | 0.80 | 0.06 | | 0.80 | | 0.76 | | 0.85 | | 0.860 | |  |
|  | HVO_NOx_ | 0.81 | 0.05 | 0.82 | 0.77 | | 0.86 | | 0.82 | 0.06 | | 0.82 | | 0.77 | | 0.87 | | 0.025 | |  |
|  | HVO_PM+NOx_ | 0.80 | 0.06 | 0.80 | 0.75 | | 0.85 | | 0.80 | 0.06 | | 0.79 | | 0.76 | | 0.86 | | 0.465 | |  |
| Z-score FEV_1_ | Filtered air | -0.74 | 0.76 | -0.44 | -1.21 | | -0.23 | | -0.68 | 0.76 | | -0.48 | | -1.16 | | -0.20 | | 0.218 | |  |
|  | HVO_NOx_ | -0.70 | 0.67 | -0.59 | -1.19 | | -0.20 | | -0.66 | 0.73 | | -0.51 | | -1.14 | | -0.04 | | 0.251 | |  |
|  | HVO_PM+NOx_ | -0.76 | 0.78 | -0.56 | -1.32 | | -0.31 | | -0.75 | 0.82 | | -0.56 | | -1.20 | | -0.28 | | 0.740 | |  |
| Z-score FVC | Filtered air | -0.44 | 0.75 | -0.24 | -0.97 | | 0.00 | | -0.40 | 0.83 | | -0.29 | | -0.89 | | 0.10 | | 0.438 | |  |
|  | HVO_NOx_ | -0.47 | 0.67 | -0.30 | -0.84 | | 0.17 | | -0.52 | 0.72 | | -0.50 | | -0.94 | | 0.23 | | 0.420 | |  |
|  | HVO_PM+NOx_ | -0.45 | 0.77 | -0.45 | -0.73 | | 0.17 | | -0.48 | 0.85 | | -0.50 | | -0.70 | | 0.35 | | 0.636 | |  |
| Z-score FEV_1_/FVC | Filtered air | -0.56 | 0.69 | -0.32 | -1.21 | | -0.10 | | -0.50 | 0.77 | | -0.49 | | -1.20 | | -0.22 | | 0.653 | |  |
|  | HVO_NOx_ | -0.43 | 0.69 | -0.40 | -0.77 | | -0.03 | | -0.28 | 0.73 | | -0.20 | | -0.93 | | 0.19 | | 0.011 | |  |
|  | HVO_PM+NOx_ | -0.60 | 0.67 | -0.49 | -1.15 | | -0.27 | | -0.55 | 0.67 | | -0.44 | | -0.95 | | -0.21 | | 0.420 | |  |
| %pred FEV_1_ | Filtered air | 90.83 | 9.50 | 94.43 | 85.60 | | 97.27 | | 91.57 | 9.45 | | 93.93 | | 86.13 | | 97.69 | | 0.218 | |  |
|  | HVO_NOx_ | 91.37 | 8.22 | 92.39 | 85.87 | | 97.44 | | 91.80 | 9.02 | | 93.80 | | 86.40 | | 99.59 | | 0.314 | |  |
|  | HVO_PM+NOx_ | 90.54 | 9.72 | 93.16 | 84.28 | | 96.34 | | 90.63 | 10.39 | | 92.73 | | 85.48 | | 96.70 | | 0.687 | |  |
| %pred FVC | Filtered air | 94.63 | 9.30 | 97.03 | 88.03 | | 100.06 | | 95.17 | 10.28 | | 96.38 | | 89.00 | | 101.28 | | 0.438 | |  |
|  | HVO_NOx_ | 94.23 | 8.29 | 96.25 | 89.48 | | 102.06 | | 93.60 | 9.00 | | 94.11 | | 88.18 | | 102.95 | | 0.420 | |  |
|  | HVO_PM+NOx_ | 94.55 | 9.56 | 94.48 | 91.18 | | 102.06 | | 94.24 | 10.51 | | 93.88 | | 91.35 | | 104.41 | | 0.653 | |  |
| %pred FEV_1_/FVC | Filtered air | 95.56 | 5.58 | 97.70 | 90.78 | | 99.37 | | 95.91 | 5.87 | | 96.59 | | 90.78 | | 98.41 | | 0.586 | |  |
|  | HVO_NOx_ | 96.53 | 5.27 | 97.02 | 94.25 | | 99.79 | | 97.63 | 5.42 | | 98.57 | | 93.02 | | 101.33 | | 0.013 | |  |
|  | HVO_PM+NOx_ | 95.29 | 5.13 | 96.47 | 91.74 | | 98.11 | | 95.71 | 5.05 | | 96.70 | | 92.86 | | 98.47 | | 0.446 | |  |

**Table 2**: The estimated mean in spirometry parameters during each exposure scenario (estimated mean), and differences between the two HVO exposures and FA exposure (beta). The beta values (L min^-1^) and significance (p-value) are based on the linear mixed model with exposure order correction. Values within brackets are the 95% CI.

|  |  | Estimated mean (95%CI) | Beta (95% CI) | P-value |
| --- | --- | --- | --- | --- |
| FEV_1_ (L) | Filtered air | 0.02 [-0.02,0.06] | Ref |  |
|  | HVO_NOx_ | 0.01 [-0.04,0.05] | -0.01 [-0.07,0.04] | 0.618 |
|  | HVO_PM+NOx_ | 0.02 [-0.03,0.06] | 0 [-0.06,0.05] | 0.868 |
| FVC (L) | Filtered air | 0.02 [-0.03,0.07] | Ref |  |
|  | HVO_NOx_ | -0.01 [-0.06,0.04] | -0.03 [-0.1,0.03] | 0.318 |
|  | HVO_PM+NOx_ | -0.02 [-0.07,0.03] | -0.04 [-0.11,0.02] | 0.195 |
| FEV_1_/FVC | Filtered air | 0 [-0.01,0.01] | Ref |  |
|  | HVO_NOx_ | 0 [0,0.01] | 0 [-0.01,0.01] | 0.695 |
|  | HVO_PM+NOx_ | 0.01 [0,0.02] | 0.01 [0,0.02] | 0.230 |
| Z-score FEV_1_ | Filtered air | 0.06 [-0.03,0.15] | Ref |  |
|  | HVO_NOx_ | 0.01 [-0.08,0.1] | -0.05 [-0.16,0.07] | 0.396 |
|  | HVO_PM+NOx_ | 0.04 [-0.05,0.12] | -0.02 [-0.14,0.09] | 0.687 |
| Z-score FVC | Filtered air | 0.04 [-0.05,0.13] | Ref |  |
|  | HVO_NOx_ | -0.03 [-0.12,0.06] | -0.07 [-0.19,0.05] | 0.247 |
|  | HVO_PM+NOx_ | -0.05 [-0.14,0.04] | -0.09 [-0.21,0.03] | 0.123 |
| Z-score FEV_1_/FVC | Filtered air | 0.06 [-0.07,0.19] | Ref |  |
|  | HVO_NOx_ | 0.05 [-0.08,0.18] | -0.01 [-0.19,0.18] | 0.942 |
|  | HVO_PM+NOx_ | 0.15 [0.02,0.27] | 0.09 [-0.09,0.27] | 0.307 |
| %pred FEV_1_ | Filtered air | 0.75 [-0.39,1.89] | Ref |  |
|  | HVO_NOx_ | 0.1 [-1.04,1.24] | -0.65 [-2.11,0.82] | 0.379 |
|  | HVO_PM+NOx_ | 0.42 [-0.69,1.54] | -0.32 [-1.77,1.12] | 0.655 |
| %pred FVC | Filtered air | 0.55 [-0.58,1.68] | Ref |  |
|  | HVO_NOx_ | -0.3 [-1.43,0.83] | -0.85 [-2.38,0.67] | 0.266 |
|  | HVO_PM+NOx_ | -0.63 [-1.74,0.47] | -1.18 [-2.69,0.32] | 0.121 |
| %pred FEV_1_/FVC | Filtered air | 0.35 [-0.6,1.29] | Ref |  |
|  | HVO_NOx_ | 0.42 [-0.53,1.36] | 0.07 [-1.27,1.4] | 0.921 |
|  | HVO_PM+NOx_ | 1.1 [0.18,2.02] | 0.75 [-0.57,2.07] | 0.259 |

**G. Baseline oscillometry values and atypical group**

**Table.** The average oscillometric parameters measured before the volunteer exposures. The oscillometric values averting from the specified limit of R_5-19_, A_X_ and X_5_ are shaded, and fulfilling ≥ 2 of these were the inclusion criteria of the “atypical” group. The volunteer who did not participate in all exposures was excluded (n=18).

|  |  |  | **R_5_** | | **R_19_** | | **R_5-19_** | | **A_X_** | | **X_5_** | | **F_res_** | |
| --- | --- | --- | --- | --- | --- | --- | --- | --- | --- | --- | --- | --- | --- | --- |
|  |  |  | **(cmH_2_O s L^-1^)** | | **(cmH_2_O s L^-1^)** | | **(cmH_2_O s L^-1^)** | | **(cmH_2_O L^-1^)** | | **(cmH_2_O s L^-1^)** | | **(Hz^-1^)** | |
| **Group** | **Patient** | **Sex** | **Avg.** | **Std. dev.** | **Avg.** | **Std. dev.** | **Avg.** | **Std. dev.** | **Avg.** | **Std. dev.** | **Avg.** | **Std. dev.** | **Avg.** | **Std. dev.** |
| Atypical criteria (≥ 2)* | | |  |  |  |  | ≥ 0.8 |  | ≥ 14 |  | ≤ -1.8 |  |  |  |
| Typical | 1 | M | 2.91 | 0.33 | 2.43 | 0.16 | 0.48 | 0.26 | 5.25 | 0.68 | -1.1 | 0.04 | 14.54 | 0.55 |
|  | 2 | M | 1.65 | 0.2 | 1.66 | 0.13 | -0.01 | 0.08 | 1.4 | 0.18 | -0.65 | 0.08 | 9.29 | 0.19 |
|  | 3 | M | 2.76 | 0.15 | 2.52 | 0.13 | 0.24 | 0.09 | 2.82 | 0.39 | -0.74 | 0.1 | 12.96 | 1.05 |
|  | 4 | M | 3.08 | 0.44 | 2.19 | 0.27 | 0.89 | 0.31 | 9.37 | 3.27 | -1.2 | 0.24 | 19.73 | 1.68 |
|  | 5 | F | 3.11 | 0.21 | 2.96 | 0.07 | 0.15 | 0.2 | 3.51 | 1.14 | -1.04 | 0.14 | 11.71 | 1.49 |
|  | 6 | M | 3.97 | 0.44 | 3.26 | 0.24 | 0.72 | 0.28 | 9.38 | 3.03 | -1.19 | 0.13 | 20.3 | 2.91 |
|  | 7 | F | 3.55 | 0.16 | 3.36 | 0.15 | 0.2 | 0.17 | 5.75 | 0.81 | -1.17 | 0.15 | 17.26 | 2.5 |
|  | 8 | M | 3.44 | 0.26 | 2.86 | 0.25 | 0.59 | 0.11 | 5.07 | 2.02 | -1.03 | 0.27 | 14.16 | 1.1 |
|  | 9 | F | 3.19 | 0.23 | 3.08 | 0.16 | 0.11 | 0.12 | 3.98 | 0.21 | -1.17 | 0.04 | 11.83 | 0.37 |
|  | 10 | F | 3.7 | 0.51 | 3.2 | 0.32 | 0.5 | 0.2 | 4.78 | 1.16 | -1.18 | 0.16 | 13.24 | 1.51 |
|  | 11 | F | 4.26 | 0.72 | 3.7 | 0.51 | 0.56 | 0.32 | 10.31 | 4.61 | -1.27 | 0.12 | 19.6 | 3.54 |
|  | 12 | F | 4.35 | 0.37 | 3.82 | 0.07 | 0.53 | 0.31 | 6.82 | 2.67 | -1.42 | 0.31 | 14.84 | 2.53 |
|  | 13 | M | 4.35 | 0.78 | 3.77 | 0.51 | 0.58 | 0.43 | 8.21 | 5.72 | -1.29 | 0.46 | 17.16 | 3.48 |
| Atypical | 14 | M | 3.84 | 0.11 | 2.5 | 0.09 | 1.33 | 0.05 | 14.86 | 0.82 | -1.33 | 0.13 | 23.59 | 0.62 |
|  | 15 | M | 7.35 | 2.21 | 3.9 | 0.86 | 3.45 | 1.41 | 42.8 | 23.41 | -4.12 | 1.59 | 26.91 | 5.85 |
|  | 16 | F | 5.69 | 0.46 | 4.45 | 0.26 | 1.24 | 0.46 | 15.41 | 5.36 | -2.05 | 0.42 | 21.19 | 3.04 |
|  | 17 | F | 4.84 | 0.22 | 3.94 | 0.13 | 0.89 | 0.1 | 14.13 | 1.8 | -1.74 | 0.17 | 23.12 | 1.83 |
|  | 18 | F | 5.15 | 0.68 | 3.51 | 0.32 | 1.64 | 0.85 | 18.68 | 8.6 | -1.91 | 0.61 | 22.84 | 1.38 |
| * The atypical criteria were based on the physician’s evaluation of the oscillogram (1) and based on demographics of normal versus asthmatic subjects in (2). | | | | | | | | | | | | | | |

**H. Study population details**

**Table.** The study population details. Breathing frequency was measured with a respiratory inductance plethysmograph (Nox T3 breathing belt, Nox Medical, ResMed) and the average tidal volume by forced oscillometric technique (FOT) (Tremoflo, THORASYS, Thoracic Medical System Inc.,) before the exposure sessions. Baseline FEV_1_ and FVC was measured by spirometry (SPIRARE 3, DIAGNOSTICA), and medical history was taken during the initial medical exam before commencement of the study.

|  | **Study subject details** | | | | **Baseline breathing pattern** | | | |  |  | **Medical history** | | |
| --- | --- | --- | --- | --- | --- | --- | --- | --- | --- | --- | --- | --- | --- |
| **ID** | **Sex** | **Age (years)** | **Height (cm)** | **Weight (kg)** | **Breathing**  **freq. (min^-1^)** | **Avg. tidal volume (L)** | **FEV_1_ (L)** | **FVC (L)** | **Exposure order group*** | **‘Atypical FOT’** | **Phadiatop pos.** | **History of symptoms and/or atopic symptoms** | **Symptoms of bronchial hyperreactivity** |
| 1 | M | 37 | 176 | 66 | 14.6 | 0.44 | 3.6 | 4.59 | 2 | no | yes | yes | - |
| 2 | M | 37 | 191 | 81 | 14.3 | 1.47 | 5.12 | 6.6 | 1 | no | yes | yes | - |
| 3 | M | 29 | 182 | 79 | 13.7 | 1.05 | 4.42 | 5.27 | 3 | no | yes | yes | Yes |
| 4 | M | 48 | 174 | 89 | 21.5 | 0.89 | 3.76 | 4.69 | 4 | no | yes | yes | - |
| 5 | F | 25 | 167 | 56 | 19.3 | 0.68 | 3.33 | 3.96 | 2 | no | yes | yes | yes |
| 6 | M | 32 | 167 | 69 | 24.9 | 1.00 | 3.4 | 3.73 | 9 | no | yes | - | - |
| 7 | F | 29 | 180 | 67 | 15.7 | 1.50 | 3.11 | 3.92 | 9 | no | - | - | - |
| 8 | M | 28 | 172 | 72 | 17.1 | 0.63 | 3.51 | 3.99 | 6 | no | - | - | - |
| 9 | F | 29 | 175 | 70 | 14.4 | 0.64 | 3.3 | 3.61 | 2 | no | - | - | - |
| 10 | F | 51 | 173 | 66 | 22.8 | 0.49 | 3.38 | 4.2 | 1 | no | - | - | - |
| 11 | F | 55 | 171 | 77 | 20.1 | 0.84 | 2.69 | 3.34 | 7 | no | - | yes | - |
| 12 | F | 21 | 164 | 65 | 15.5 | 0.54 | 3.2 | 3.57 | 3 | no | - | - | - |
| 13 | M | 25 | 160 | 69 | 19.3 | 0.86 | 3.35 | 3.67 | 1 | no | - | yes | yes |
| 14 | M | 28 | 178 | 80 | 16.5 | 1.18 | 3.89 | 5.07 | 5 | yes | yes | yes | - |
| 15 | M | 35 | 176 | 71 | 16.6 | 1.32 | 2.82 | 3.22 | 6 | yes | - | yes | - |
| 16 | F | 48 | 163 | 69 | 18.3 | 1.08 | 2.58 | 3.51 | 2 | yes | - | yes | yes |
| 17 | F | 27 | 163 | 85 | 21.1 | 0.82 | 3.32 | 4.02 | 8 | yes | - | - | - |
| 18 | F | 21 | 166 | 79 | 17.1 | 0.44 | 3.26 | 3.96 | 2 | yes | - | yes | yes |
| 19 | M | 20 | 181 | 94 | 13.3 | 0.98 | 4.96 | 5.77 | 7 | no | - | - | - |
| * Exposure group order detailed in additional file I | | | | | | | | | | | | | |

**I. Exposure order**

**Table.** The exposure order and number of subjects (n) in each exposure order group (Additional File H). All participants went through a short mock-up of the exposure order session during baseline medical exams and the imbalanced exposure order was accounted for by the statistical analysis.

| **Exposure order group** | **Exposure order** | **n** |
| --- | --- | --- |
|  | **(min. 1 week wash-out in between)** |  |
| 1 | HVO_NOx_ – HVO_PM+NOx_ – FA – Salt | 3 |
| 2 | HVO_NOx_ – Salt – HVO_PM+NOx_ – FA | 5 |
| 3 | Salt – FA - HVO_NOx_ - HVO_PM+NOx_ | 2 |
| 4 | HVO_NOx_ – FA – Salt – HVO_PM+NOx_ | 1 |
| 5 | FA – HVO_NOx_ – HVO_PM+NOx_ – Salt | 1 |
| 6 | FA – HVO_NOx_ – Salt – HVO_PM+NOx_ | 2 |
| 7 | HVO_NOx_ – Salt – FA – HVO_PM+NOx_ | 2 |
| 8 | HVO_PM+NOx_ – HVO_NOx_ – FA – Salt | 1 |
| 9 | Salt – HVO_NOx_ – HVO_PM+NOx_ – FA | 2 |

**References**

1. Lundblad LKA, Siddiqui S, Bossé Y, Dandurand RJ. Applications of oscillometry in clinical research and practice. Can J Respir Crit Care, Sleep Med. 2019;0(0):1–15. https://doi.org/10.1080/24745332.2019.1649607

2. Eddy RL, Westcott A, Maksym GN, Parraga G, Dandurand RJ. Oscillometry and pulmonary magnetic resonance imaging in asthma and COPD. Physiol Rep. 2019;7(1):1–12.

3. Asgharian B, Hofmann W, Bergmann R. Particle deposition in a multiple-path model of the human lung. Aerosol Sci Technol. 2001;34(4):332–9.

4. Rissler J, Swietlicki E, Bengtsson A, Boman C, Pagels J, Sandström T, et al. Experimental determination of deposition of diesel exhaust particles in the human respiratory tract. J Aerosol Sci. 2012;48:18–33.
